# Transcriptome analysis of *Pantoea rara* mutants reveals the underlying complexity of bacterial phosphate solubilization

**DOI:** 10.1128/spectrum.03363-25

**Published:** 2025-12-15

**Authors:** Holly Hone, Tongda Li, Jennifer L. Wood, Jatinder Kaur, Timothy Sawbridge

**Affiliations:** 1 Agriculture Victoria, AgriBio, Centre for AgriBioscience 124398 , Bundoora, Victoria, Australia; 2 DairyBio, AgriBio, Centre for AgriBioscience 124398 , Bundoora, Victoria, Australia; 3 School of Applied Systems Biology, La Trobe University, Bundoora, Victoria, Australia; 4 Department of Microbiology, Anatomy, Physiology and Pharmacology, La Trobe University, Bundoora, Victoria, Australia; University of Minnesota Twin Cities, St. Paul, Minnesota, USA

**Keywords:** phosphate solubilization, biofertilizer, mutation, bacterial transcriptome, AICAR, histidine biosynthesis, acidolysis

## Abstract

Biofertilizers offer a promising avenue to reduce phosphate fertilizer reliance. However, inconsistent lab-to-field conversion undermines confidence in the technology. Increasingly, initial screens of bacteria are conducted *in silico,* based on the presence of a widely accepted set of phosphate solubilization-associated “canon” genes. However, these genes capture only a subset of the mechanisms observed experimentally in microbe-mediated solubilization. This study uses a transcriptomic systems approach to compare the transcriptional response of three novel *Pantoea rara* strains with divergent phosphate solubilization indices (PSI): a wild type (PSI = 1.74), an enhanced efficiency mutant (PSI = 4.13), and a null mutant (PSI = 0), which are indistinguishable using canonical gene markers alone, to phosphate limitation. Soluble phosphate limitation triggered robust but transient transcriptional responses across 80 Kyoto Encyclopedia of Genes and Genomes (KEGG) pathways. Despite its presence, the *gcd* gene, the most commonly used marker for phosphate solubilization functionality, was not upregulated in either of the solubilizing strains. Instead, each strain appeared to rely on a unique combination of organic acids, including acetic, citric, formic, and malic acid. Notably, the divergent phenotypes of both mutants were linked to a rewiring of the phosphate limitation response, namely a heightened limitation response in the enhanced mutant and an apparent “phosphate blindness” in the null mutant. These findings suggest that system-level characterization of phosphate solubilizers can reveal auxiliary pathways and genes overlooked by current annotation frameworks. Integration of auxiliary contributors with the existing canon could improve the predictive power of pre-field phosphate solubilization screens and subsequently help bridge the lab-to-field gap in biofertilizer development.

**IMPORTANCE:**

Despite the potential benefits to the productivity of agricultural systems and the health of local ecosystems, enthusiasm for phosphate-solubilizing biofertilizers has been dampened by a significant performance gap that occurs when candidates are transitioned from the lab to the field. This inconsistency is partially attributable to narrow screening strategies that rely on simplified *in vitro* assays and the broad use of a narrow set of canonical genes, such as *gcd*, as functional proxies. This study leveraged a rare set of three *Pantoea rara* strains that differ phenotypically in their phosphate solubilization capacity but are identical using standard genetic markers. This system enabled the identification of overlooked genetic and regulatory contributors. These findings reveal limitations in current screening methods and underscore the need for a more comprehensive molecular framework to guide biofertilizer discovery.

## INTRODUCTION

The environmental toll and economic burden of synthetic fertilizer production are significant. The production, transportation, and application of fertilizers account for approximately 5%–6% of total greenhouse gas emissions (1, 2). The environmental impacts of these emissions are compounded by the ecological consequences of nutrient runoff from improper fertilization regimes (3, 4). Furthermore, for farmers, steadily rising fertilizer prices, which are projected to increase by 0.8%–3.6% annually until 2050, place significant pressure on profit margins (5, 6). These challenges, alongside legislated reductions in usage, create an urgent need for sustainable, affordable alternatives (7–10). Biofertilizers, microbes able to improve plant growth by enhancing nutrient availability and uptake from the environment, have become the focus of significant research and commercial interest (11–13). However, many biofertilizers that demonstrate promising results in controlled laboratory conditions underperform in the field (14–16). This disconnect, which has led to some skepticism about the industry as a whole, is commonly attributed to (i) misalignment between the biofertilizer strain and the target agroecosystem, and (ii) limitations in existing pre-field screening methods (13, 17–19). Both issues stem, at least in part, from the inherent complexity of formulating universal strategies to harness dynamic, multi-dimensional biological systems. The success of biofertilizers is, therefore, dependent on the development of more comprehensive, ecologically informed standards for selection and application.

The nutritional contribution of a biofertilizer to its target crop is influenced by climate, edaphic conditions, host genotype, and the endemic microbiome (20–22). The complexity of identifying a non-local strain with a niche that overlaps with the specific requirements of the target agroecosystem creates a myriad of opportunities for misalignment (13). However, as long as the microbial ecology of the target agroecosystem remains intact, plant growth promoters capable of facilitating greater nutrient uptake, antagonistic biocontrol of phytopathogens, abiotic stress tolerance, and plant growth can often be identified within the existing microbial ecosystem (12, 23–26). As a result, researchers have begun to mine the native microbiome of target crop species, in a “personalized medicine” or “host-mediated” approach (13). This approach integrates plant genetics, microbial population dynamics, and environmental context to prioritize ecological considerations and ensure compatibility with the host system (17, 27, 28). As such, there is growing interest in seed microbiomes. Seed microbiomes show evidence of host curation, or microbial resources tailored to the genotype, developmental stages, geography, and environmental pressures (29–32).

Once isolated, the axenic assessment of a candidate biofertilizer’s capacity and mode of action is challenging, given the limitations of existing pre-field screening methods (13, 18, 33). This is particularly acute for phosphate solubilizers, which, unlike their nitrogen-fixing counterparts, remain comparatively understudied (34, 35). The identification of phosphate solubilizers typically involves two methods, used either independently or in combination: *in vitro* media screening using tricalcium phosphate, and *in silico* functional prediction based on a limited subset of phosphate solubilization-associated genes (33, 34). These methods, while useful in high-throughput screens, operate on simplified models of phosphate solubilization that do not capture the complexity of microbe-mediated phosphate solubilization *in situ*. Tricalcium phosphate, for example, is much easier to solubilize than the phosphate forms that dominate the soil (e.g., hydroxyapatite, aluminum/iron phosphate, or orthophosphate) and is, therefore, a controversial universal selection factor, despite its ubiquity (18, 36). Similarly, the “canonical” gene set, associated with the production of gluconic acid (*gcd*, *pqqBCDE*) and common drivers of enzymolysis (*appA, acpD, phoA*), represents only a fraction of the phosphate solubilization mechanisms observed experimentally (33, 37). Seven key solubilization strategies have been experimentally identified: (i) acidolysis via organic acid secretion; (ii) enzymolysis via the secretion of enzymes, such as phosphatase; (iii) redox-mediated solubilization; (iv) chelation-driven mineral dissolution; (v) H+ excretion via ammonium assimilation; (vi) extracellular oxidation via the direct oxidation pathway; and (vii) exopolysaccharide-associated mobilization (37–45). Research on the metabolic processes upstream and downstream of these mechanisms is still limited, and the related genes have not been identified (34, 46). Even in the case of acidolysis, the phosphate solubilization mechanism that is perhaps best understood, mediated by *gcd,* is an inappropriate universal marker as it is less common in Gram-positive microbes, which instead favor other kinds of organic acid (39, 47–49). As a result, the genetic “canon” is limited in its ability to reliably predict phosphate solubilization (50). Transcriptomics analysis facilitates a system-wide interrogation of the activity of known phosphate solubilizers, thereby supporting the identification of auxiliary systems and genes (51–54). In combination with the current genetic canon, these auxiliary genes may improve the ability to predict phosphate solubilization capacity in candidate biofertilizers, helping to bridge the lab-to-field gap in biofertilizer development (12, 17, 55).

In Australia, the nitrogen needs of lucerne pastures are already predominantly met by the industry standard rhizobium, *Ensifer meliloti* RRI128 (56). A system-appropriate phosphate solubilizer could reduce the chemical inputs required to meet lucerne’s most pressing nutritional need (30, 50, 56). In a previous study, host curation was leveraged to identify a seed-associated phosphate solubilizer, Gram-negative *Pantoea rara* Lu_Sq_004, from *Medicago sativa* cv. Sequel (50). Functional assessment based on the limited set of “canonical” genes assessed by *in silico* screens seemed to indicate that the high phosphate solubilization activity of *P. rara* Lu_Sq_004 on Pikovskaya (PVK) medium was largely due to gluconic acid production, with potential support from secreted enzymes (50). Yet, by the same criteria, an enhanced-efficiency mutant P+ (phosphate solubilization indices [PSI] = 4.13) and null mutant P− (PSI = 0) appear functionally identical, as their distinguishing mutations lie outside the canonical gene set (50).

This study had three aims. First, to examine whether the presence of members of the genic canon corresponds to their active employment in phosphate solubilization on PVK agar. Second, to compare the transcriptomes of wild-type and UV-generated mutants to identify the “auxiliary” genes, currently overlooked, whose disruption causes significant variation of the phosphate solubilization phenotype. Finally, to elucidate the genetic basis for enhanced phosphate solubilization.

## MATERIALS AND METHODS

### Isolate source

*P. rara* Lu_Sq_004, isolated from *Medicago sativa* cv. Sequel seed demonstrated high-efficiency phosphate solubilization (PSI = 1.74) using Pikovskaya agar (PVK, per liter: yeast extract, 0.5 g; dextrose, 10 g; calcium phosphate, 5 g; ammonium sulfate, 0.5 g; potassium chloride, 0.2 g; magnesium sulfate, 0.1 g; manganese sulfate, 0.0001 g; ferrous sulfate, 0.0001 g; and agar, 15 g.) (18, 57). An enhanced function mutant (Lu_Sq_004_1_2 or P+) and null mutant (Lu_Sq_004_6_5 or P−) were generated using UV mutagenesis using an Ultra-Lum UV Crosslinker (UVAC, 230 V, 50 HZ, 1 A) (50, 58). Details on the isolation and mutation of these strains are available in Supplementary 1.

### Sample generation

A transcriptomic experiment was designed to capture expression changes resulting in the variance in the phosphate solubilization index. Wild-type Lu_Sq_004 and its mutant derivatives were retrieved from −80°C glycerol storage, inoculated onto R2A plates, and incubated at room temperature for 5 days. Single colonies were plated in four replicates on both R2A and PVK plates. There were three plates per treatment. Plates were harvested every 2 days over a 10-day growth period. To harvest, the four replicate colonies on each plate were scraped into DNA/RNA Protection Reagent (Zymo Research) and stored at −80°C (59, 60). Total RNA was extracted using a Total RNA Miniprep Kit (T2010, NEB), rRNA depleted (E7860, NEB), and libraries were prepared using the Ultra II Directional RNA Library Prep Kit (E7765, NEB). The resulting library was sequenced on an Illumina NovaSeq X platform by a commercial service provider (Australian Genome Research Facility, Melbourne, Australia). RNA-seq data (raw reads) were quality-trimmed and filtered using fastp, and residual rRNA sequences were removed using bbmap (61).

### Transcriptome analysis

Transcript abundance was estimated using Salmon, with a reference transcriptome generated from the Prokka-annotated genome of Lu_Sq_004_WT (62). Reads were quantified using Salmon using the following parameters: -l A, --numBootstraps 1000, --seqBias, and –numBiasSamples 5000000. Differential gene expression analysis was conducted using Sleuth with salmon-generated transcript abundances (63). Low-abundance transcripts were filtered using a custom function requiring transcripts to have ≥3 estimated counts in at least 47% of samples. Statistical significance of sample group separation observed in the principal coordinate analysis (PCoA) was assessed using PERMANOVA using vegan (64). Likelihood ratio tests and Wald tests were performed to identify differentially expressed transcripts (q ≤ 0.05). Overlapping sets of significant transcripts from both tests were extracted to identify robust differentially expressed gene (DEG) candidates. Significant DEGs were used to generate heatmaps with ComplexHeatmap (65). Kyoto Encyclopedia of Genes and Genomes (KEGG) pathway enrichment analysis was conducted using clusterProfiler with KEGG pathway annotations derived from EggNOG. Adjusted *P* values ( false discovery rate [FDR]-corrected) were used to identify the pathways that were significantly enriched when the bacterial transcriptome profiles on PVK and R2A were compared (66). Results were visualized using ggplot2 (Supplementary 2 to 6) (67).

## RESULTS

### Soluble phosphate limitation drives transcriptional changes in Lu_Sq_004_WT and its mutants

**Fig 1:**
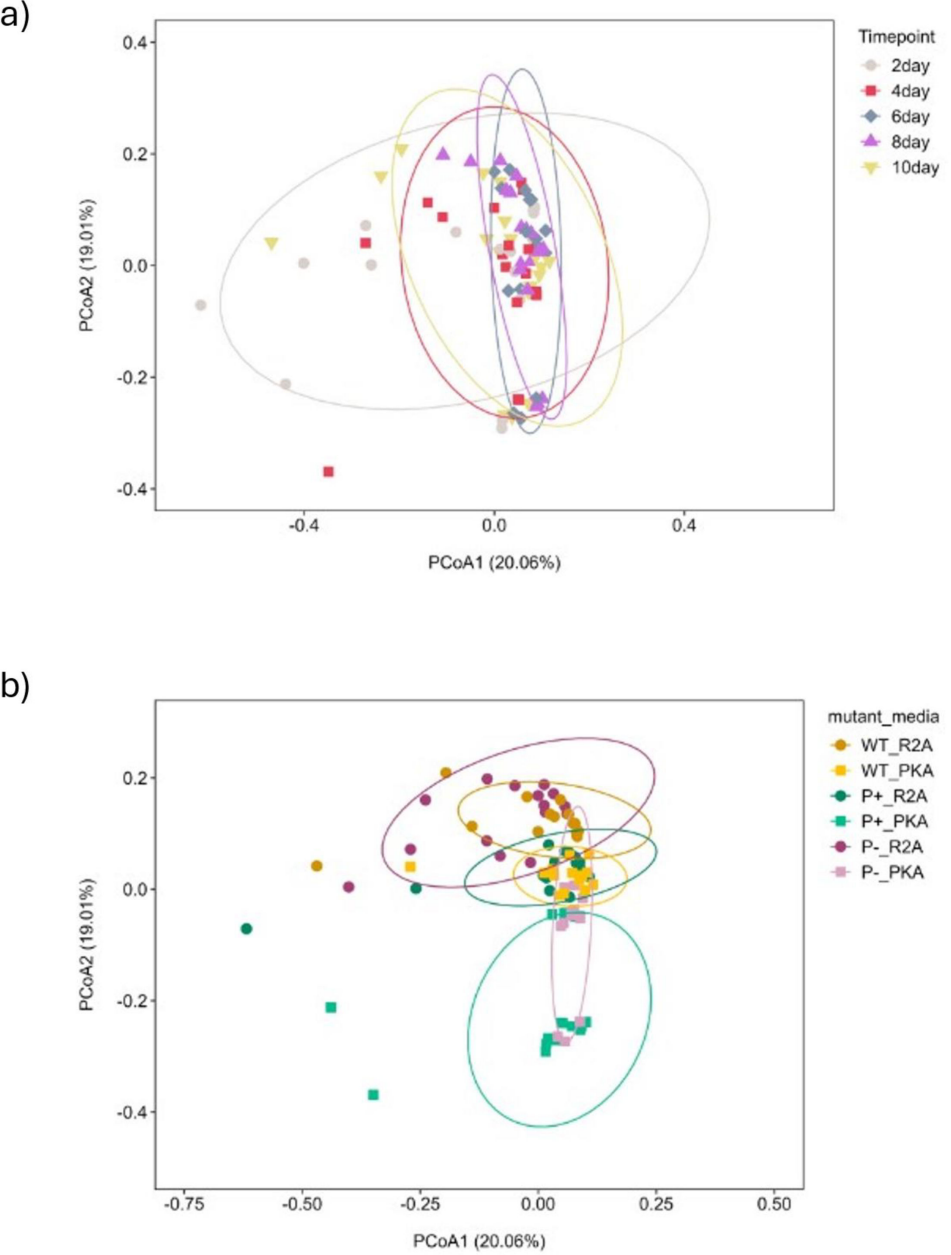
(**a**) Principal coordinates analysis based on Bray-Curtis dissimilarity reveals transcriptional shifts between time points. PCoA was performed on transcriptomic profiles of *P. rara* wild-type and mutant strains across five time points (days 2, 4, 6, 8, and 10). Each point represents a biological replicate (*n* = 9 per group), with shape and color indicating time point (i.e., gray circle = 2 day, red square = 4 day, blue diamond = 6 day, purple triangle = 8 day, and yellow inverted triangle = 10 day). Ellipses represented 95% confidence intervals. (**b**) Principal coordinates analysis based on Bray-Curtis dissimilarity reveals transcriptional shifts between media. PCoA was generated on transcriptomic profiles of *P. rara* wild-type and mutant strains grown under LSP and RSP. Each point represents a biological replicate (*n* = 15 per group), with shape indicating media (e.g., circle = R2A, square = PVK), color representing strain (orange = WT, green = P+, purple = P−). Ellipses represented 95% confidence intervals.

**Fig 2:**
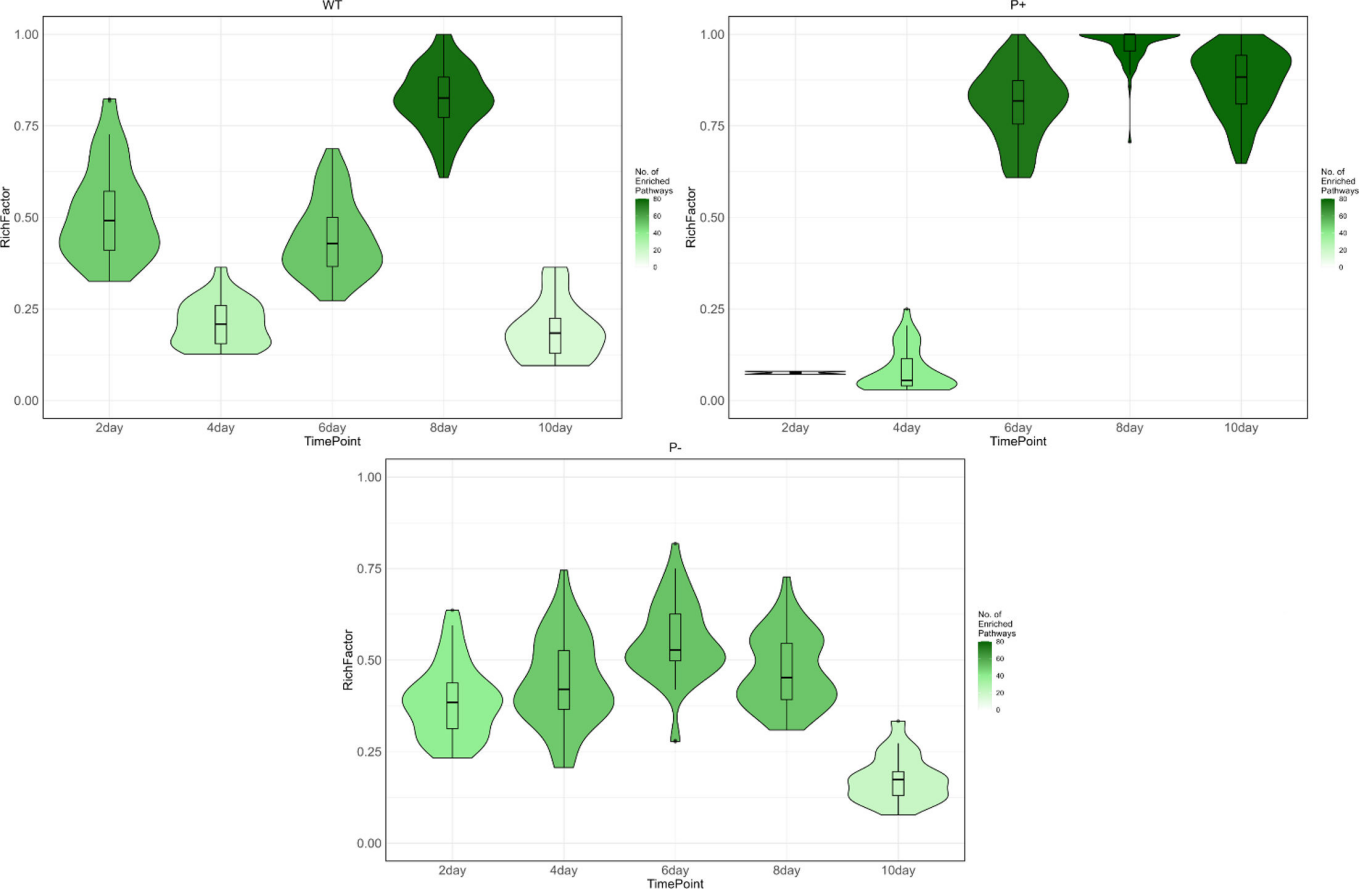
Violin-overlaid boxplots showing the distribution of KEGG pathway enrichment (RichFactor) across time points for each strain (*P. rara* WT, enhanced mutant P+, and null mutant P−). Boxplots and violins are colored according to the number of enriched pathways detected at each time point; a green gradient scale (light = fewer pathways, dark = more pathways) reflects the depth of pathway enrichment, facilitating comparison between time points and strains.

**Fig 3:**
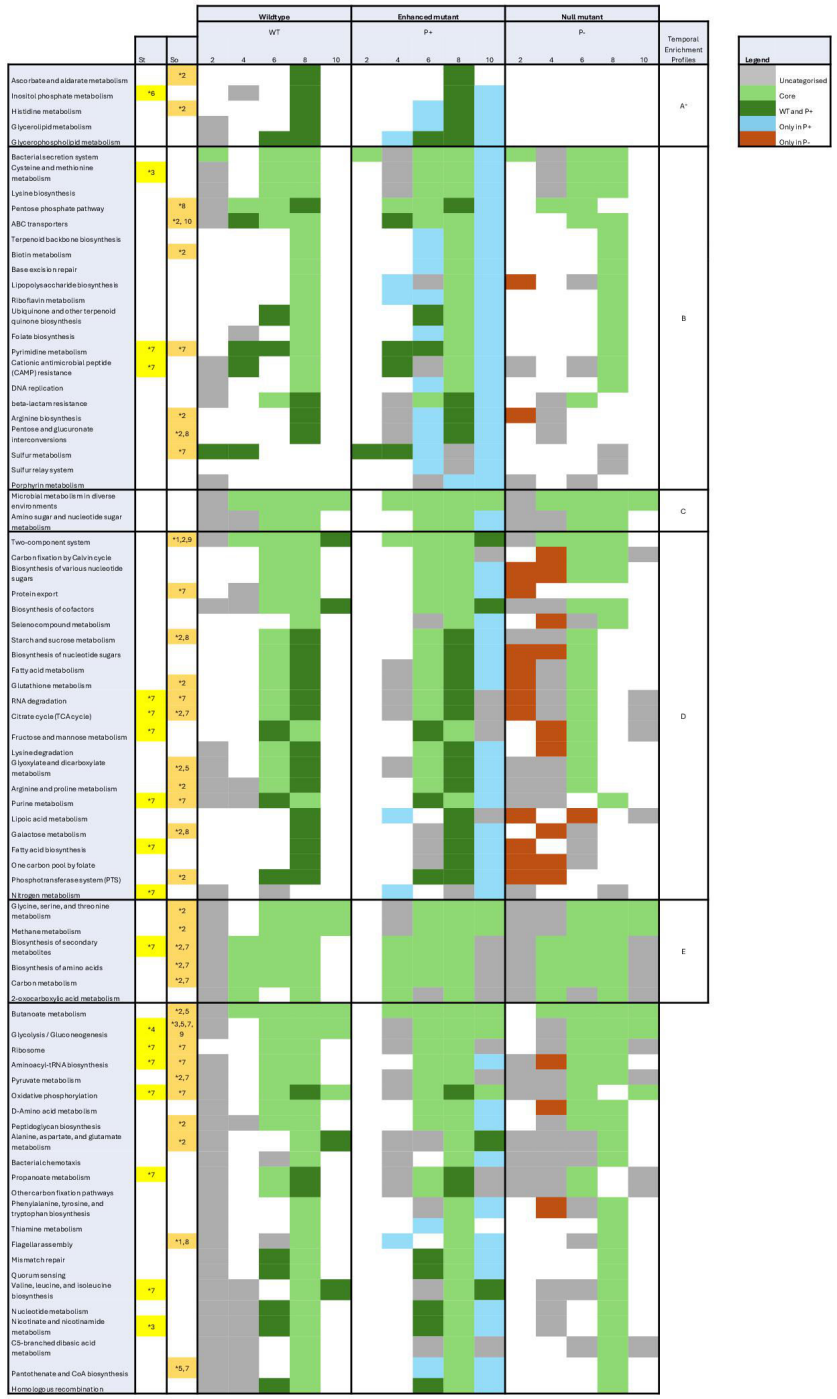
Temporal KEGG pathway enrichment profiles between media across *P. rara*. Each cell represents the enrichment status of a given pathway in a specific strain at a given time point. Colored cells represent a significantly enriched pathway. KEGG pathways were grouped based on their temporal enrichment profile. Pathway previously associated with phosphate starvation is highlighted in yellow in the St column, and phosphate solubilization is highlighted in orange in So. *1. (68) 2. (52) 3. (69) 4. (70) 5. (71) 6. (72) 7. (53) 8. (73) 9. (74) 10. (75) ^+^Given the solubilizer-exclusive pathway enrichment, found in group A pathways, is most likely to reflect mechanistic relevance, expression differences within these KEGG pathways were visualized (Supplementary 8 to 11). Differential gene expression in histidine metabolism under LSP, compared to RSP, is described in “3.3.1 Enhanced phosphate solubilization mutant.”

Transcriptome profiles of high-efficiency Lu_Sq_004_WT, an enhanced efficiency mutant, P+, and a non-solubilizing null mutant, P−, grown on PVK and R2A, were analyzed over time. Bray-Curtis dissimilarity-based principal coordinates analysis showed significant variation between the replete phosphate media (R2A) and limited phosphate media (PVK) across time (Fig. 1). The first principal coordinate (PCoA1) accounted for 20.06% of total variance. Separation along PCoA1 was influenced by the harvest time point, with bacterial profiles from 2, 4, and 10 days after inoculation tending to fall to the left of the x-axis, while samples from days 6 and 8 clustered together on the right of the x-axis (Fig. 1a, 2, and 3). The second principal coordinate (PCoA2) accounted for 19.01% of the total variance. Separation along PCoA2 appears to be predominantly driven by media conditions, with R2A profiles (replete soluble phosphate, RSP) higher on the y-axis than corresponding PVK profiles (limited soluble phosphate, LSP) for each microbe (Fig. 1b). PERMANOVA analysis confirmed the separation between the transcriptome of each strain differed significantly when exposed to the two media conditions, which explained variance (R^2^) among significant comparisons (threshold of *P* = 0.001), ranging from 13% to 28% (Supplementary 7). The only transcriptome profiles that did not differ when separated by mutant and media were those of wild-type and null mutant on R2A.

### KEGG pathway enrichment analysis between media conditions

Pathway enrichment analysis identified underlying shifts in transcriptional profiles across time points. KEGG pathway enrichment analysis identifies biological pathways where differentially expressed genes are statistically overrepresented. Figure 2 visualizes the temporal shifts in RichFactor for the WT, the enhanced solubilization mutant, and the null-phosphate solubilization mutant when grown on PVK compared to R2A. RichFactor represents the proportion of DEGs within each pathway impacted by treatment, in this case, soluble phosphate availability (66). There were distinct differences in how the RichFactor varied over time between WT and mutant strains. In the WT, the proportion of DEGs within significantly enriched pathways peaked on day 8, with significant differences between other time steps (Fig. 2). In contrast, there was almost no significant enrichment in the enhanced mutant on days 2 and 4; however, by day 6, almost all genes within enriched pathways were differentially expressed. The RichFactor remained high for the enhanced mutant on days 8 and 10. The RichFactor of the null phosphate solubilizer rose steadily from day 2, peaking at 0.55 on day 4 and declining by day 10.

Significant pathway enrichment across the five time points was grouped based on their temporal enrichment profiles (Fig. 3). Eighty pathways were significantly enriched in at least one strain at least one recorded time point. The number of core pathways, defined as pathways enriched by all three microbes at the same time point, highlighted the transcriptomic shifts that were conserved across strains, despite their variable phosphate solubilizing capacity when exposed to varying levels of soluble phosphate. The number of pathways increased steadily from day 2 (1) to day 8 (50), then declined by day 10 (6), seeming to demonstrate a cascade of metabolic responses in response to phosphate limitation. There were five temporal enrichment profiles. Group A contained five pathways enriched in phosphate solubilizers (wild type and enhanced), but never in the null phosphate solubilization mutant. Ascorbate and aldarate metabolism, histidine metabolism, and inositol phosphate metabolism have been previously associated with either phosphate starvation or phosphate solubilization (52, 72). Group B contained 21 pathways, which were more consistently enriched in the enhanced function mutant than in either the wild-type or null phosphate solubilization mutant. Group C contained two pathways, which had uniform enrichment in the wild-type and null phosphate solubilization mutant, but unique temporal enrichment in the enhanced function mutant. Group D contained 23 pathways, which showed, simultaneously, earlier enrichment in the null phosphate solubilization mutant and delayed enrichment in the enhanced mutant, compared to the wild type. Group E contained six pathways, which had more consistent enrichment in the null phosphate solubilization mutant compared to both the phosphate solubilizers.

### Mutational impact on “canonical” phosphate solubilization gene expression

**Fig 4:**
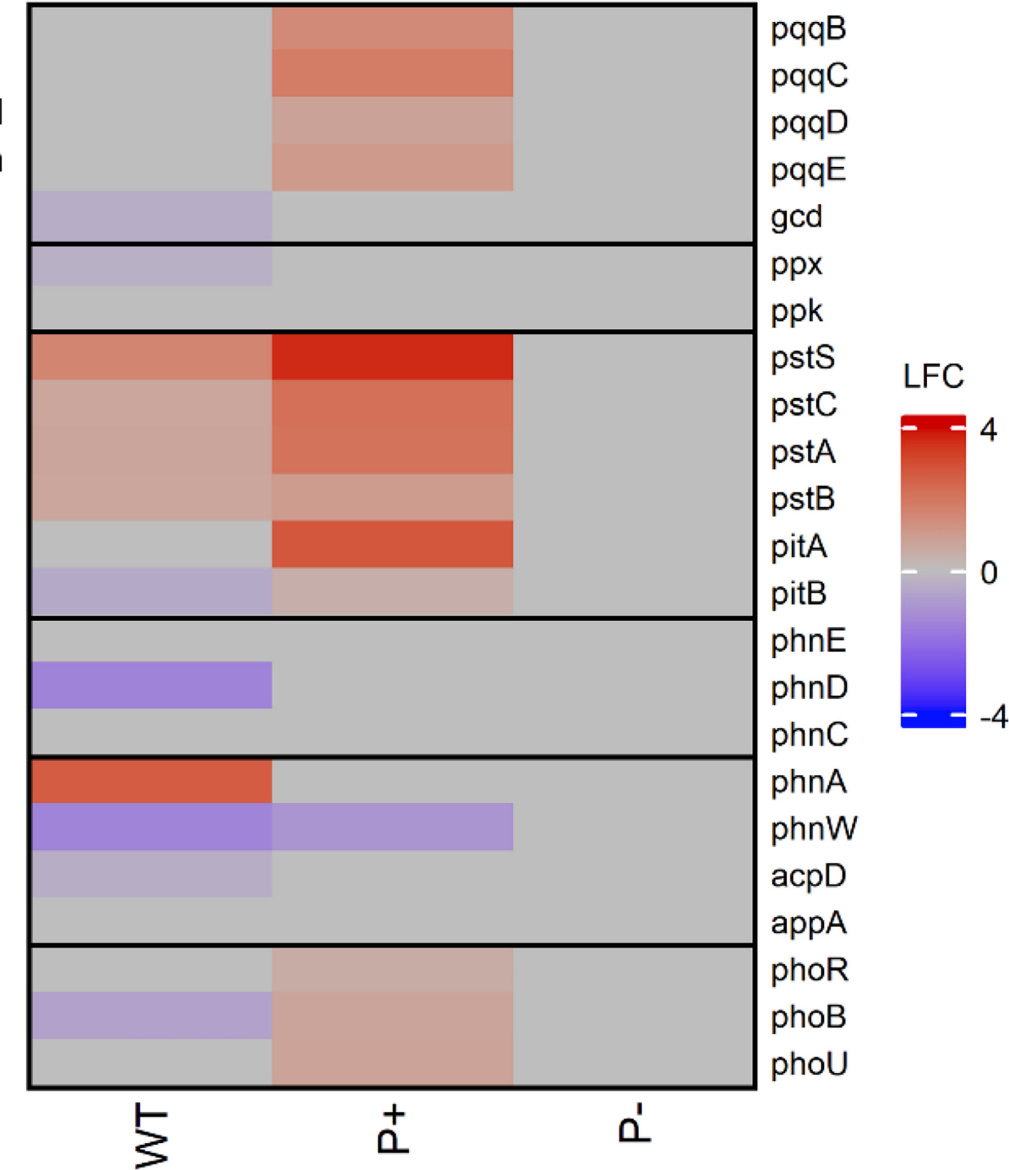
Heatmaps of DEGs associated with key phosphate solubilization on day 8. Pairwise comparison of phosphate solubilization DEGs in each strain on differing media, with RSP (R2A) as the baseline. Each cell represents a Wald test-derived normalized log_2_ fold change in expression (LFC) of a given gene relative to its expression on R2A.

*In silico* functional analysis of *P. rara* WT suggested that the isolate had the capacity to secrete both gluconic acid and a fleet of enzymes (phytase, phosphatase, and C-P lyase) to solubilize recalcitrant forms of environmental phosphate (50). The genes governing these mechanisms were examined to elucidate the wild-type baseline and expression differences in the phosphate solubilization mutant. Day eight was selected for DEG comparison as it had the highest number of “core” enriched pathways (Fig. 3) and the highest RichFactor for phosphate solubilizers (Fig. 4). Both phosphate solubilizers showed significant upregulation of the high-affinity inorganic phosphate transporter operon, *pstSCAB*. In the wild type, there was an upregulation of *phnA* (log_2_ fold change, LFC = 2.67) but a downregulation of *phnW* (LFC = −1.48), *acpD* (LFC = −0.42), and *ppx* (LFC = −0.37). There was also a downregulation of low-affinity inorganic phosphate transporter *pitB*, organic phosphate transporter *phnD*, and regulator *phoB*. Conversely*, pstSCAB*, *pitAB*, *phoBR*, and *phoU* were exclusively upregulated in the enhanced phosphate solubilization mutant. The only phosphate solubilization gene downregulated in the enhanced mutant was enzymolysis-associated *phnW*. None of the key phosphate solubilization genes were differentially expressed under LSP conditions in the null phosphate solubilization mutant. Notably, *gcd*, which produces glucose hydrogenase, was not upregulated in either of the phosphate solubilizers. In fact, *gcd* was downregulated in the wild type on day 8 (LFC = −0.42).

**Fig 5:**
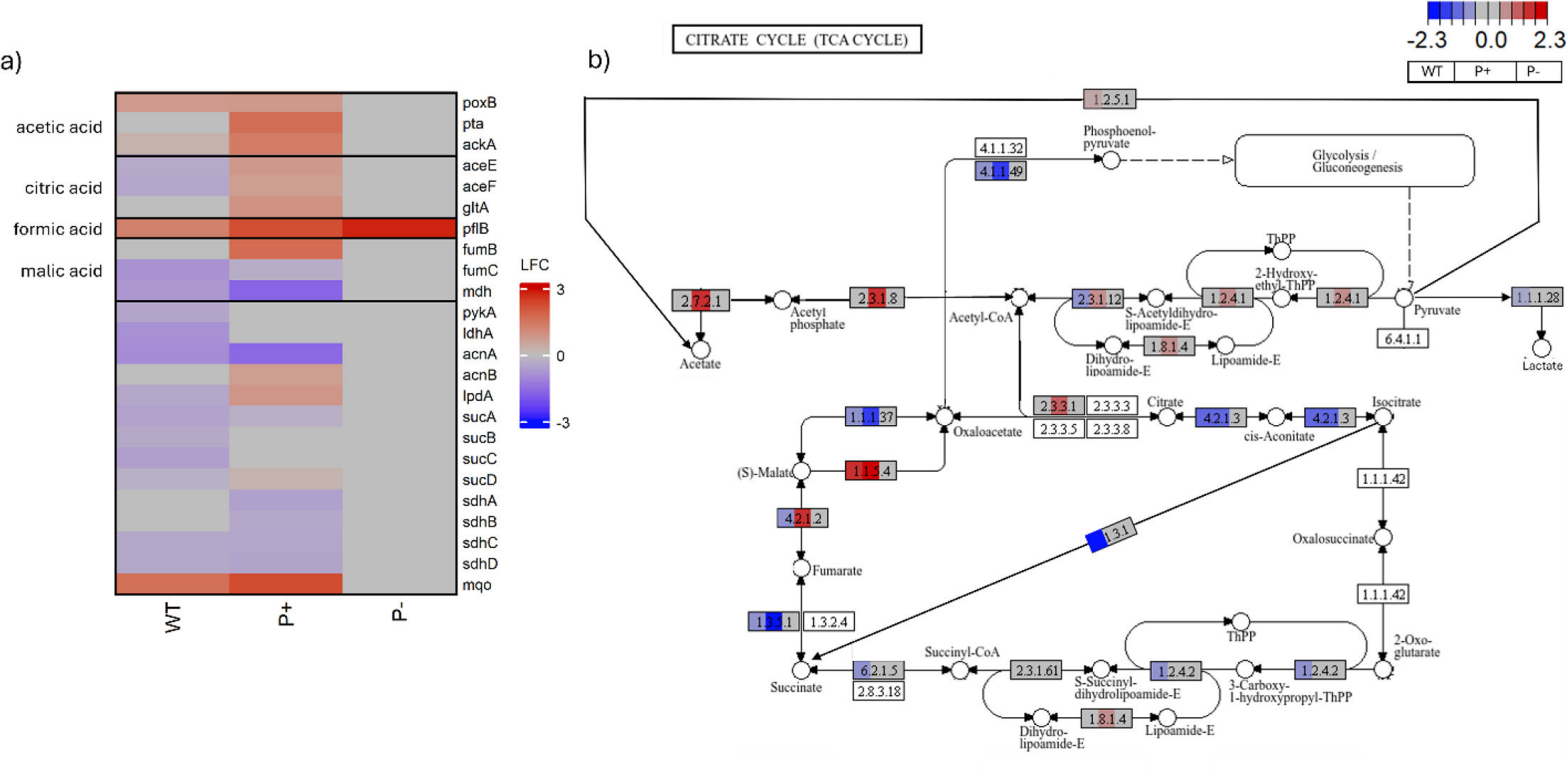
(**a**) Heatmaps of DEGs associated with organic acid production on day 8. Pairwise comparison of organic acid DEGs in each strain on differing media, with RSP (R2A) as the baseline. Each cell represents Wald test-derived normalized log_2_ fold change in expression (LFC) of a given gene relative to its expression on R2A. (**b**) Modified KEGG tricarboxylic acid (TCA) cycle pathway overlaid with differential gene expression profiles of *P. rara* wild type and mutant, with RSP (R2A) as the baseline, displayed with gene expression fold change mapped onto the pathway nodes. Each gene node in the KEGG pathway is colored according to Wald test-derived LFC (WT in the first third, P+ in the middle third, P− in the last third) in expression of that gene between LSP (PVK) and RSP (R2A) at day 8. Only genes that passed a false discovery threshold of q <0.05 are colored; nonsignificant changes appear unshaded or gray. Enzymes encoded by the genes depicted in the heatmap are *aceE*—1.2.4.1/*aceF*—2.3.1.12 pyruvate dehydrogenase multienzyme complex; *acnA/B*—4.2.1.3 aconitase; *fumB/C*—4.2.1.2 fumarase; *gltA*—2.3.3.1 citrate synthase; *aceA*—4.1.3.1 isocitrate lyase; *ldhA*—1.1.1.28 d-lactate dehydrogenase; *mdh*—1.1.1.37 malate dehydrogenase; *mqo*—1.1.5.4 malate dehydrogenase; *pckA*—4.1.1.49 phosphoenolpyruvate carboxykinase; *poxB*—1.2.5.1 pyruvate dehydrogenase; *pta*—2.3.1.8/*ackA*—2.7.2.1 phosphate acetyltransferase/acetate kinase; *ptsG*, a phosphotransferase system enzyme; *pykA*—4.1.1.49 pyruvate kinase; *sfcA*, malic enzyme; *sdhABCD*—1.3.5.1 succinate dehydrogenase; *sucA*—1.2.4.2/*sucB*—2.3.1.61/*lpdA*—1.8.1.4—ketoglutarate dehydrogenase complex; *sucC/D*—6.2.1.5 succinyl-CoA synthetase.

With no evidence of *gcd* expression under LSP conditions, genes associated with the production of alternative organic acids were also considered (42, 76). In the wild type, only three organic acid genes, two associated with acetic acid production (*ackA* [LFC = 0.26] and *poxB* [LFC = 0.77]) and one associated with formic acid (*pflB* [LFC = 1.3]), were upregulated (Fig. 5a and b) (77, 78). The *mqo* gene, associated with oxaloacetate accumulation, was also upregulated (79). In the enhanced function mutant, there was significant upregulation of acetic acid genes *poxB* (LFC = 0.79), *pta* (LFC = 1.67), and *ackA* (LFC = 1.41)*,* formic acid genes *pflB* (LFC = 2.23), and citric acid genes *gltA* (LFC = 0.93) (77, 78, 80).

### Impact of UV-induced point mutations on soluble phosphate limitation responses

#### *hisG* drives phosphate-dependent elevated histidine biosynthesis in the enhanced mutant

**Fig 6:**
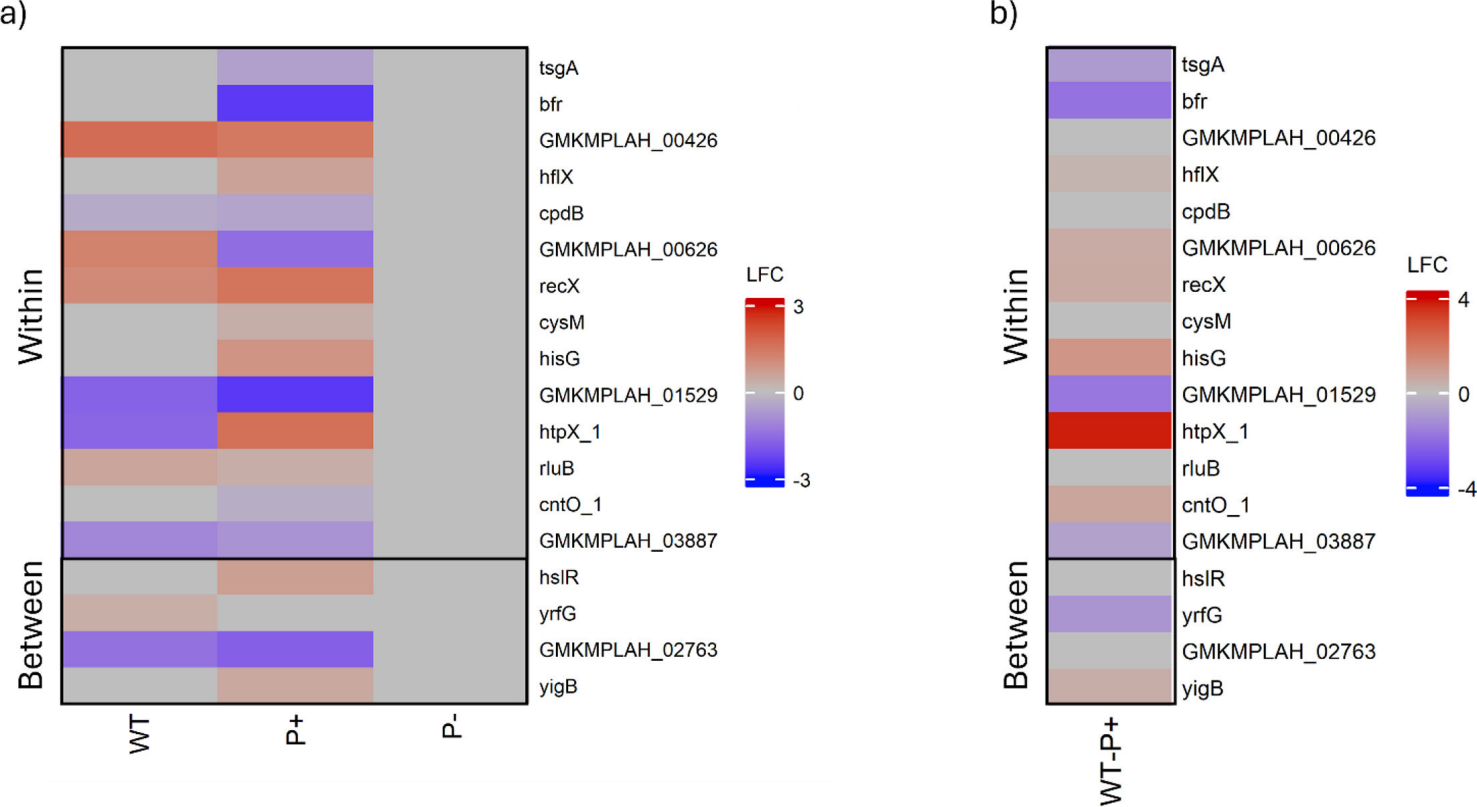
Heatmaps of DEGs associated with UV-induced point mutations in *P. rara* enhanced mutant, P+. Heatmaps display the expression profiles of genes directly affected by UV-induced point mutations (within genes) and genes adjacent to intergenic mutations (between genes) on day 8. (**a**) Pairwise comparison of enhanced mutant DEGs in each strain on differing media. Each cell represents the Wald test-derived normalized log_2_ fold change in expression (LFC) of a given gene relative to its expression on R2A. (**b**) Pairwise comparison of enhanced-mutant DEGs of WT and P+ on PVK, with wild type as the baseline. Each cell represents the normalized LFC of a given gene relative to its expression in WT.

Differential abundance analysis was used to investigate the expression of the 24 unique point mutations in the enhanced mutant P+ identified in previous research (50). Notably, none of these genes showed differential expression under LSP in the null phosphate solubilization mutant. A pairwise comparison of the wild-type and enhanced mutant under RSP to their own expression profiles under LSP on day 8 allowed direct comparison of the transcriptomic impact of the UV-induced point mutations (Fig. 6a).

Genes GMKMPLAH_00426, *cpdB, cysM, rluB, hslR*, GMKMPAH_02763, although differentially expressed when the bacterial transcriptomes under RSP and LSP at the same time point were compared, show no significant differences in expression when the wild-type and enhanced mutant were compared on PVK (soluble phosphate limitation) (Fig. 6b). The *hisG, htpX_1, hflX, yigB, cntO_1, recX,* and GMKMPLAH_00626 genes were upregulated in the enhanced mutant, compared to the wild type. Notably, *hisG* codes for an ATP phosphoribosyltransferase, which acts as the first step of histidine metabolism, was one of the five group A KEGG pathways (Fig. 3) that were exclusively expressed in phosphate solubilizers when exposed to LSP (Table 1) (81).

**TABLE 1:** Differential expression in point mutation-affected genes in the enhanced phosphate solubilization mutant (P+) under LSP on day 8, compared to the wild type (as the baseline) under the same conditions

Predicted gene name	Mutation location relative to gene	LFC	KO	KEGG pathway
*htpX_1*	Within	3.81	K03799	–^*a*^
*hisG*	Within	1.16	K00765	Histidine metabolism, biosynthesis of secondary metabolites, biosynthesis of amino acids
*cntO_1*	Within	0.71	K02014	–
*recX*	Within	0.57	K03565	–
GMKMPLAH_00626	Within	0.54	–	–
*yigB*	Between	0.48	K20862	Riboflavin metabolism, biosynthesis of secondary metabolites, biosynthesis of cofactors
*hflX*	Within	0.28	K03665	–
GMKMPLAH_03887	Within	−0.73	–	–
*tsgA*	Within	−0.93	K06141	–
*yrfG*	Between	−1.05	K20881	Purine metabolism, nucleotide metabolism
GMKMPLAH_01529	Within	−1.80	–	–
*bfr*	Within	−1.94	K03594	Porphyrin metabolism

^*a*^ – denotes fields unpopulated by KEGG.

Both *htpX_1* (a zinc-dependent membrane) and *hflX* (an ATP-dependent RNA helicase) code for heat shock proteins (82, 83). *yigB* codes for a flavin mononucleotide phosphatase, a crucial step in the biosynthesis of riboflavin (84). Riboflavin metabolism (group B, Fig. 3) was more consistently expressed in the enhanced function mutant than in either the wild-type or null phosphate solubilization mutant (Table 1). The *cntO_1* gene codes for a TonB-dependent receptor associated with the uptake of siderophores, vitamin B12, and saccharides (85). Finally, *recX* is an important negative regulator of RecA, a key factor in SOS response activation, DNA repair, and recombination (86).

Conversely, *bfr, yrfG*, *tsgA,* GMKMPLAH_03887*,* and GMKMPLAH_01529 were significantly downregulated under LSP compared to the wild type. Bacterioferritin, encoded by *bfr* and part of porphyrin metabolism (group B, Fig. 3), regulates the storage and utilization of iron, which is essential for the growth and metabolism of microorganisms (87). The *yrfG* gene codes for a GMP/IMP nucleotidase, specifically cytosolic 5′-nucleotidase II, which plays a crucial role in purine metabolism by catalyzing the hydrolysis of purine monophosphates (88). The purine metabolism KEGG pathway (group D, Fig. 3) showed early enrichment from the null phosphate solubilization mutant, followed by staggered enrichment from the two phosphate solubilizers, in order of efficiency. The *tsgA* gene codes for an MFS transporter (Fucose:H+ symporter family), involved in transporting substrates across cell membranes (89).

**Fig 7:**
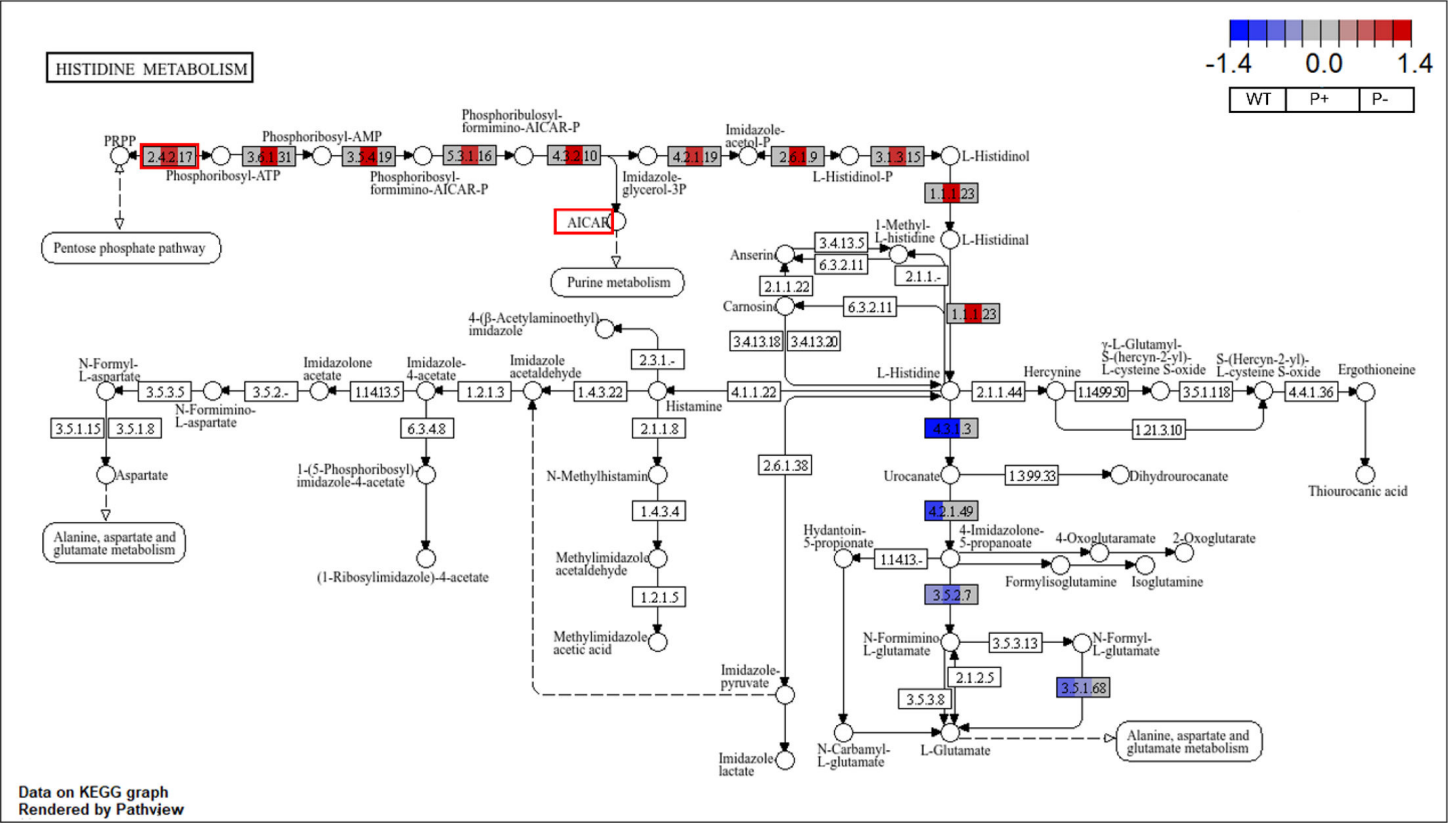
KEGG histidine metabolism pathway overlaid with differential gene expression profiles of *P. rara* wild-type and mutant strains, with RSP (R2A) as the baseline. The KEGG histidine metabolism pathway (map00340) is displayed with gene expression fold change mapped onto the pathway nodes, highlighting how histidine-associated genes are transcriptionally modulated in response to phosphate conditions and UV-induced mutations. Each gene node in the KEGG pathway is colored according to Wald test-derived LFC (WT in the first third, P+ in the middle third, P− in the last third) in expression of that gene between LSP and RSP at day 8. The mutated *hisG* gene is highlighted in red.

Figure 7 shows differential gene expression of wild-type, enhanced, and null mutants under LSP. The downstream effects of the mutant-*bfr* gene and the mutant-*yrfG* gene on the purine metabolism pathways and porphyrin metabolism, respectively, were unclear. However, the upregulation of mutant-*hisG* appears in concert with the downstream upregulation of

- *hisI* (EC:3.5.4.19 + 3.6.1.31, LFC = 1.36)
- *hisA* (EC:5.3.1.16, LFC = 0.87)
- *hisF* (EC:4.3.2.10, LFC = 1.11)
- *hisB* (EC:4.2.1.19 + 3.1.3.15, LFC = 1.05)
- *hisC* (EC:2.6.1.9, LFC = 1.24)
- *hisD* (EC:1.1.1.23, LFC = 1.30)

in the enhanced phosphate solubilization mutant. There was no differential expression in the his operon in either the wild-type or null phosphate solubilization mutant in response to LSP.

#### Phosphate variation does not alter gene expression in null mutant

**Fig 8:**
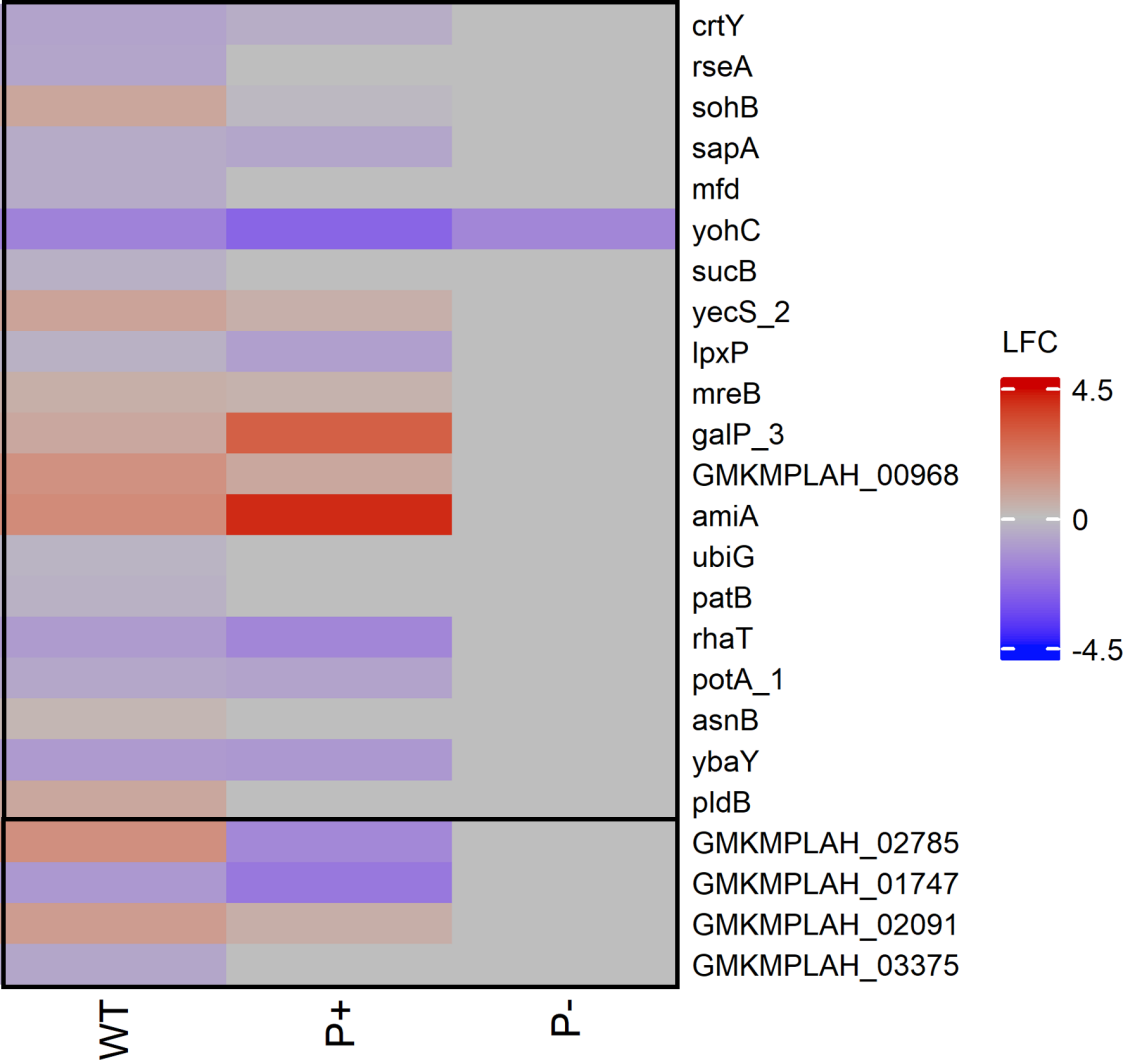
Heatmaps of DEGs associated with UV-induced point mutations in *P. rara* null mutant, P−, with RSP (R2A) as the baseline. Heatmaps display the expression profiles of genes directly affected by UV-induced point mutations (within genes) and genes adjacent to intergenic mutations (between genes) on day 8. Pairwise comparison of null mutant DEGs in each strain on differing media. Each cell represents the Wald test-derived normalized log_2_ fold change in expression (LFC) of a given gene relative to its expression on R2A.

P− demonstrated an inability to produce a halo on PVK after 10 days of growth despite no point mutations within key “canon” phosphate solubilization genes (50). The transcriptome of the null mutant shows clear shifts in pathway enrichments compared to the wild type, but a complete lack of differential expression in the phosphate solubilization canon (Fig. 3 and 4). Medaka analysis identified a total of 69 point mutations; 14 located in intergenic spaces and 55 located within annotated genes (50). Nineteen mutated genes and five of the genes located in proximity to intergenic mutations were differentially expressed when pairwise comparisons of transcriptomes of each microbe under LSP and RSP on day 8 were compared (Fig. 8). Only one gene, *yohC*, was differentially expressed under LSP in the null phosphate mutant. GMKMPLAH_02747 (LFC = 1.09), although not differentially expressed in the null mutant when a pairwise comparison between RSP and LSP conditions, was the only gene differentially expressed when the null mutant was compared to the wild type under LSP. All genes listed in Fig. 8 were expressed.

## DISCUSSION

This study uses transcriptomic systems analysis to clarify the mechanistic differences between three phenotypically divergent *P. rara* strains that are indistinguishable using canonical markers, with the aim of improving the predictive resolution of phosphate solubilization screens. Soluble phosphate availability triggered robust but transient transcriptomic shifts across 80 KEGG pathways. Although the same pathways were often enriched across strains, their expression followed distinct temporal trajectories. The phosphate solubilization phenotype was characterized by three transcriptional features: upregulation of acidolysis pathways, differential regulation of phosphate sensing and homeostatic genes, and the exclusive enrichment of five KEGG pathways. In the enhanced efficiency mutant, mutation of the *hisG* gene drove upregulation of nine downstream histidine biosynthesis genes, marking it as a likely driver of the increased phosphate solubilization phenotype. In contrast, the null mutant exhibited no differential expression in genes associated with phosphate sensing, homeostasis, or solubilization, despite soluble phosphate limitation. This lack of response to phosphate stress suggests a possible phosphate “blindness” phenotype in the null mutant, although the underlying mechanism remains unclear. Contrary to the prevailing wisdom that the presence of *gcd* equates to gluconic acid-mediated solubilization, transcriptomic data from *P. rara* suggested acidolysis was instead mediated by alternative organic acids. Further interrogation of the molecular basis of these divergent phenotypes, and subsequently the underlying solubilization mechanisms, could be achieved by integrating proteomic and metabolomic analyses.

### Phosphate solubilization capacity delays a “limitation cascade”

Incorporating temporal transcriptomics in this study revealed responses to phosphate availability that were strain-specific, pathway-level, and often ephemeral (90, 91). The complexity associated with the collection and analysis of global gene expression across multiple time points often restricts the understanding of traits to expression snapshots (90–92). Yet, transience in key phosphate starvation gene expression, organic acid production, and phosphate solubilization efficiency has been individually observed over time in multiple microorganisms (42, 93–96). In this study, 23 pathways (group D, Fig. 3) exhibited a distinct temporal separation, with pathway enrichment occurring first in the null mutant and last in the enhanced P solubilization mutant. Both the two-component system, containing the PhoBR regulon, and the TCA cycle, a significant contributor to organic acid secretion, follow this temporal separation.

Approximately half of the enriched KEGG pathways (43) have been associated with either phosphate starvation or solubilization responses in previous studies (Table 1). The common denominator between these microorganisms and *P. rara* was central carbon metabolic flux, typified by an upregulation of the TCA cycle and amino acid metabolism, in response to soluble phosphate limitation (51–53, 68). The combination of common and seemingly unique enriched pathways suggests an interplay between species-dependent transcriptional responses and conserved metabolic strategies in response to environmental phosphate (33, 34, 93). These strain-specific, ephemeral expression patterns highlight the limitation of snapshot-based or marker-focused approaches in capturing the full complexity of phosphate solubilization responses.

### Sensing scarcity: internal regulatory networks shape phosphate responses

The maintenance of phosphate homeostasis during extracellular limitation involves the PstSCAB-PhoU complex, which integrates high-affinity transport with environmental sensing, and a two-component Pho system, which orchestrates downstream transcriptional responses (97–99). The transcriptional activity of this seven-gene system, and therefore the internal assessment of phosphate availability, differed significantly between the two phosphate solubilizers. In the wild type, the expression profile spoke to a switch from active solubilization to a more passive scavenging behavior. The downregulation of PhoB, which directly or indirectly regulates over 400 proteins, contrasts with typical phosphate-stress responses, suggesting that by day 8, the wild type had largely alleviated phosphate limitation (Fig. 4) (70, 100, 101). Furthermore, the upregulation of the *pst* operon in concert with the downregulation of *ppx* suggests that solubilized Pi is scavenged from the media and sequestered as polyP (70, 93, 94). Comparatively, in the enhanced mutant, the significant upregulation of all seven genes responsible for phosphate homeostasis represents the expected response to Pi scarcity (68, 70, 95, 97). As the enhanced mutant’s PSI is 2.4-fold higher than the wild-type strain, the persistent upregulation of phosphate homeostasis suggests that mutation has amplified the internal response to phosphate availability. As the PstSCAB-PhoU-PhoBR system is unchanged between strains, the divergent expression patterns suggest the influence of auxiliary regulators, capable of rewiring the Pi scarcity response and, subsequently, phosphate solubilization.

Disparate auxiliary systems of Pi regulation have been reported across multiple bacterial genera; however, their underlying mechanisms remain uncharacterized (99, 102). Parallel Pho-independent regulation, capable of inducing broad metabolic shifts that prioritize mobilization of alternative phosphate sources and exploitation of endogenous phosphate reservoirs, has been observed in *Bacillus subtilis* and *Caulobacter crescentus* (102–104). The activity of the canonical Pho regulon can also be subject to internal modulation. This modulation can occur either through direct influence on PhoBR activation, as observed with teichoic acid, or through alterations to Pho box transcription, as seen with alarmones like (p)ppGpp (97, 105). Although the underlying mechanism varies, these auxiliary systems seem to function as the intracellular Pi signaling mechanism known to be missing from the current understanding of the PstSCAB-PhoU-PhoBR model (95, 98).

Histidine biosynthesis and inositol phosphate metabolism, KEGG pathways that were exclusively enriched in the phosphate-solubilizing *P. rara* strains, have both been associated with Pi regulation via intracellular phosphate signaling (69, 106, 107). Although genes carrying point mutations in the enhanced mutant demonstrated variance in expression, only the mutant *hisG* gene, located in the histidine biosynthesis pathway, influenced pathway-level changes. Nine genes downstream of *hisG* were upregulated under Pi limitation, including the *hisF*-mediated conversion of PRFAR to imidazole acetol-phosphate, which produces 5-aminoimidazole-4-carboxamide ribonucleotide (AICAR) as a by-product (Fig. 7) (81, 108). Excessive accumulation of AICAR, a key by-product of histidine biosynthesis, has been associated with the regulation of the PhoBR regulation in *E. coli* (81). Although the mechanism by which this regulation is enacted in bacteria is unknown, in fungal models, AICAR has been reported to promote expression of PHO genes, allowing the switch from partial to full activation under Pi limitation (69, 107). In the case of excessive accumulation, AICAR has also been observed to induce PHO genes even under replete Pi conditions (98, 107, 109). This aligns with the observation that enhanced mutant exhibits an amplified Pi scarcity response not only on PVK, but also the separation of the enhanced and the wild-type transcriptome profiles under RSP. Finally, AICAR-mediated PHO regulation is believed to occur by the inhibition of isopentenyl phosphate (IP8), a product of the inositol phosphate metabolism pathway and known suppressor of the PHO operon, and by stabilizing transcription factors Pho4 and Pho2 (106, 107, 110). While the mode of action in bacteria is likely to diverge mechanistically, it appears that AICAR acts through a functionally analogous pathway to modulate expression of the PHO regulon. The exclusive pathway enrichment, the *hisG* mutation-driven regulation, and the regulatory role of AICAR-linked rewiring of the Pi limitation response as a compelling model for the enhanced phosphate solubilization phenotype.

The lack of differential expression in the null mutant across both solubilization and phosphate homeostasis genes considered in this study suggests an insensitivity to the absence of soluble phosphate (97–99, 111). Although none of the point mutations appeared to cause differential expression in the affected genes, the impact of mutation was visible at the pathway level. Post-transcriptional regulation, metabolic rewriting, or mutational buffering could explain the disconnect between gene-level expression, pathway enrichment, and phenotype (112). This pattern could reflect a failure in transcriptional responsiveness, where the null mutant is unable to activate key regulatory programs when subjected to phosphate stress. Across both mutants, solubilization capacity hinges on the regulation mechanisms designed to detect and respond to Pi scarcity. These findings reinforce the idea that phosphate solubilization capacity is not solely determined by static markers, but is instead a function of dynamic, multilayered regulatory systems which are not captured by current annotation frameworks.

### Environmental engineering: the external secretion of solubilization agents

*In silico* functional annotation assumes that, given limited soluble phosphate conditions and the presence of the requisite carbon and nitrogen sources, phosphate-solubilizing bacteria containing *gcd* will produce gluconic acid as a primary solubilization mechanism (19, 73, 76, 113–116). The downregulation of *gcd*, which encodes glucose dehydrogenase, in the wild type and the lack of differential expression in the enhanced mutant under limited soluble phosphate conditions were therefore unexpected (Fig. 4). The downregulation of *gcd* suggests that, if acidolysis was, in fact, the mechanism in play in *P. rara* Lu_Sq_004, then it must be facilitated by an alternate organic acid. The complexity of evaluating the vast array of organic acids implicated in phosphate solubilization is likely partially responsible for gluconic acid’s status as a proxy for acidolysis. However, as organic acid production is heavily dependent on strain and environmental substrates, it is possible to narrow down the most likely candidates based on genomic and environmental context (34, 68, 96, 117).

Previous experimentation has confirmed the ability of *Pantoea* species to employ acetic, citric, formic, and malic acid alongside solubilizing tricalcium phosphate on media containing the same carbon (glucose) and nitrogen (ammonium sulfate) sources as those used in this study (96, 118, 119). Genes linked to the production of acetic acid (*poxB, pta, ackA*), citric acid (*aceE, aceF, gltA*), formic acid (*pflB*), and malic acid (*fumB, fumC, mdh*) were all present in the genome of Lu_Sq_004 (42, 77, 78, 80, 120). The wild type appeared to have upregulated the production of only acetic acid, while the enhanced mutant upregulated genes which were associated with multiple organic acids (Fig. 5a). This is consistent with previous reports in *Pantoea* that acetic acid, even when produced in concert with other organic acids, was the primary driver of phosphate solubilization (34, 96, 118). Even under field conditions, acetic acid was recovered at levels four times higher than gluconic acid in the rhizosphere of *Pantoea*-inoculated wheat, despite the *Pantoea* strain being selected based on the presence of gluconic acid-associated gene *pqqE* (118). The strain-dependent plasticity in organic acid production highlights the need to look beyond canonical markers like *gcd*, toward a more nuanced understanding of solubilization as a function of environmental context, genomic potential, and metabolic regulation.

The suppression of genes associated with enzymolysis, demonstrated in both phosphate solubilizers, is perhaps unsurprising. Phosphatase and phytase, though occasionally known to act on tricalcium phosphate, are predominantly responsible for the solubilization of organic phosphate forms (43, 44, 121–123). In the absence of their substrate of choice, the downregulation of *acpD* and lack of differential expression in the *appA* gene are in line with expectations (Fig. 4) (44, 121). Notably, as C-P lyases are unable to solubilize tricalcium phosphate, we would expect the downregulation of both *phnW* and *phnA* (43, 124). The upregulation of phosphoacetase (*phnA*) may appear to buck this trend; however, this may be part of a shift toward acetic acid production as its substrate releases acetate and inorganic phosphate (124, 125).

### Conclusion

This study reveals that the phosphate solubilization mechanisms employed by three ecologically relevant *P. rara* strains are both dynamic and complex, a direct contradiction of the picture painted by canonical gene markers. Contrary to functional prediction, comparative transcriptomics revealed a strain-specific employment of alternative organic acids, such as acetic, citric, and malic acid, rather than gluconic acid. The focus on gluconic acid in functional genomics likely reflects both its ubiquity and the analytical convenience it affords; however, its exclusive use risks obscuring the nuance of microbe-mediated acidolysis. The divergent phenotypes in the two solubilization mutants appeared to derive from a rewired phosphate limitation response. The enhanced mutant exhibited a heightened phosphate limitation response, seemingly due to a sustained intracellular Pi depletion signal mediated by AICAR overproduction. Similarly, the unchanged expression of both phosphate homeostatic and solubilizing genes despite soluble phosphate limitation suggests phosphate “blindness” in the null mutant, although the underlying mechanism is unclear. These findings emphasize the value of system-level characterization, which allows movement beyond static gene-centric approaches to capture the complex interplay of conserved and strain-specific phosphate solubilization mechanisms. The integration of the auxiliary genes and pathways identified in these investigations with the widely accepted genic canon may improve the predictive power of pre-field screens, allowing for greater lab-to-field conversions and more successful biofertilizer development. The deployment of these efficacious biofertilizers will be a key component in the reduction of chemical fertilizer usage and the transition to more sustainable farming systems.

## Data Availability

Sequences were deposited under BioProject accession number PRJNA1287267. The sequenced Lu_Sq_004_WT genome is available at https://www.ncbi.nlm.nih.gov/datasets/genome/GCA_051136265.1/.

## SUPPLEMENTAL MATERIAL

Tables S1 to S11 spectrum.03363-25-s0001.pdf Tables on strains, bubble plots, and extra KEGG pathways.

ASM does not own the copyrights to Supplemental Material that may be linked to, or accessed through, an article. The authors have granted ASM a non-exclusive, world-wide license to publish the Supplemental Material files. Please contact the corresponding author directly for reuse.
