# Supplementary material for: Transcriptome analysis of Pantoea rara mutants reveals the underlying complexity of bacterial phosphate solubilization
Source: Microbiol Spectr. 2025 Dec 15;14(2):e03363-25. doi: 10.1128/spectrum.03363-25 (PMC12889132; doi:10.1128/spectrum.03363-25)

Pantoea rara mutants reveal the underlying complexity of bacterial phosphate solubilization

Supplementary 1: Description of Lu_Sq_004 strains used in this study

In-article identifier	Strain lab name	Isolation source	Mutation method	Phenotype	Deposited	Accession
WT	Lu_Sq_004_WT	*Medicago sativa* cv. Sequel seed		PSI=1.74	National Measurement Institute (Port Melbourne, Victoria, Australia) under accession number V24/010669	CP194942
P+	Lu_Sq_004_1_2		UV exposure	PSI=4.13	National Measurement Institute (Port Melbourne, Victoria, Australia) under accession number V24/010670	
P-	Lu_Sq_004_6_5		UV exposure	PSI=0		

Unofficial

Supplementary 2: KEGG Pathway Enrichment in *Pantoea rara* wildtype and mutant strains on Day 2. The bubble plot shows the results of KEGG pathway enrichment analysis for differentially expressed genes (DEGs) the three strains when PVK and R2A are compared. Pathways are plotted on the y-axis and the x-axis indicates the RichFactor. Bubble size reflects the number of DEGs mapped to the pathway and the bubble colour indicates the statistical significance of enrichment (p-adjust <0.05).

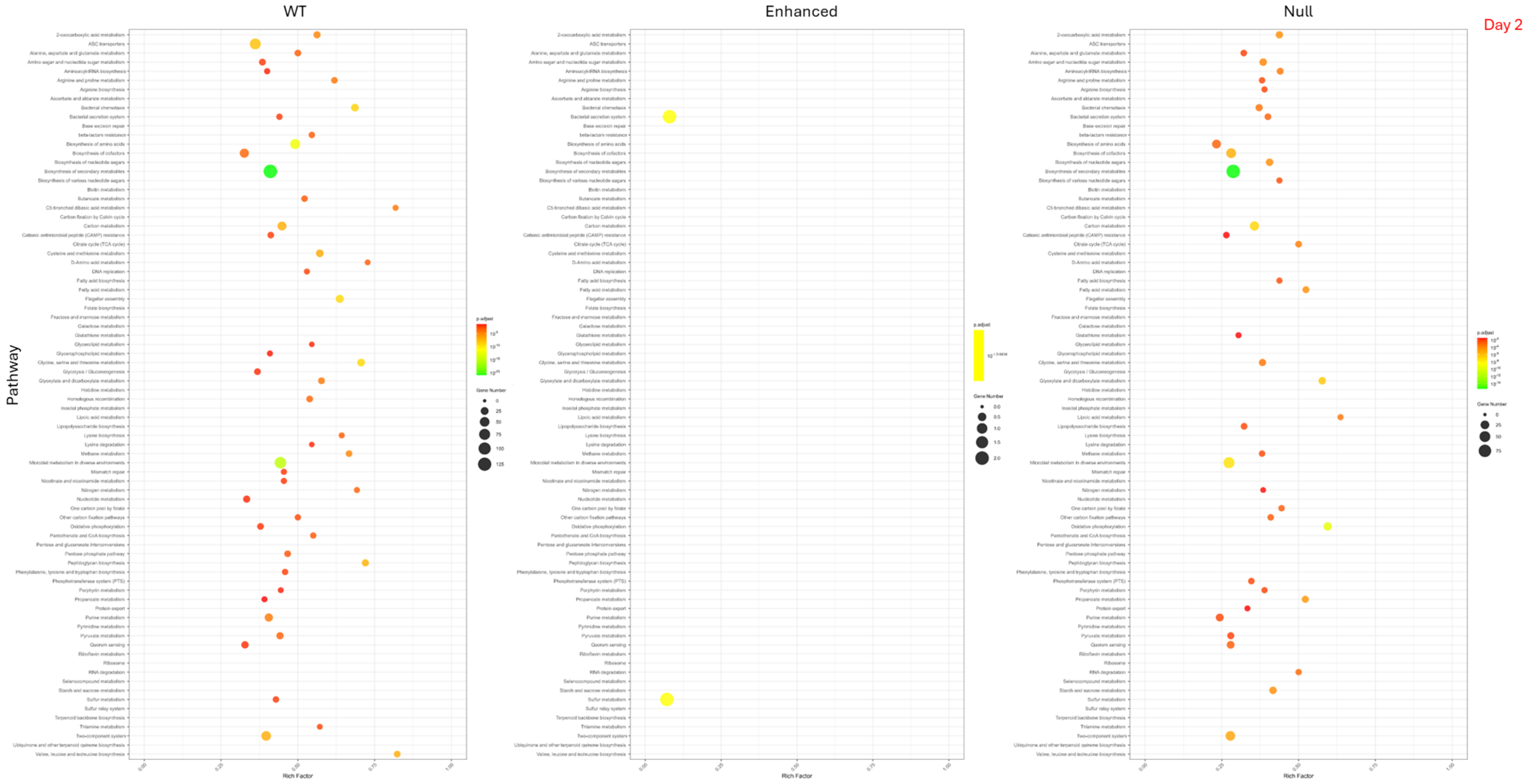

Unofficial

Supplementary 3: KEGG Pathway Enrichment in *Pantoea rara* wildtype and mutant strains on Day 4. The bubble plot shows the results of KEGG pathway enrichment analysis for differentially expressed genes (DEGs) the three strains when PVK and R2A are compared. Pathways are plotted on the y-axis and the x-axis indicates the RichFactor. Bubble size reflects the number of DEGs mapped to the pathway and the bubble colour indicates the statistical significance of enrichment (p-adjust <0.05).

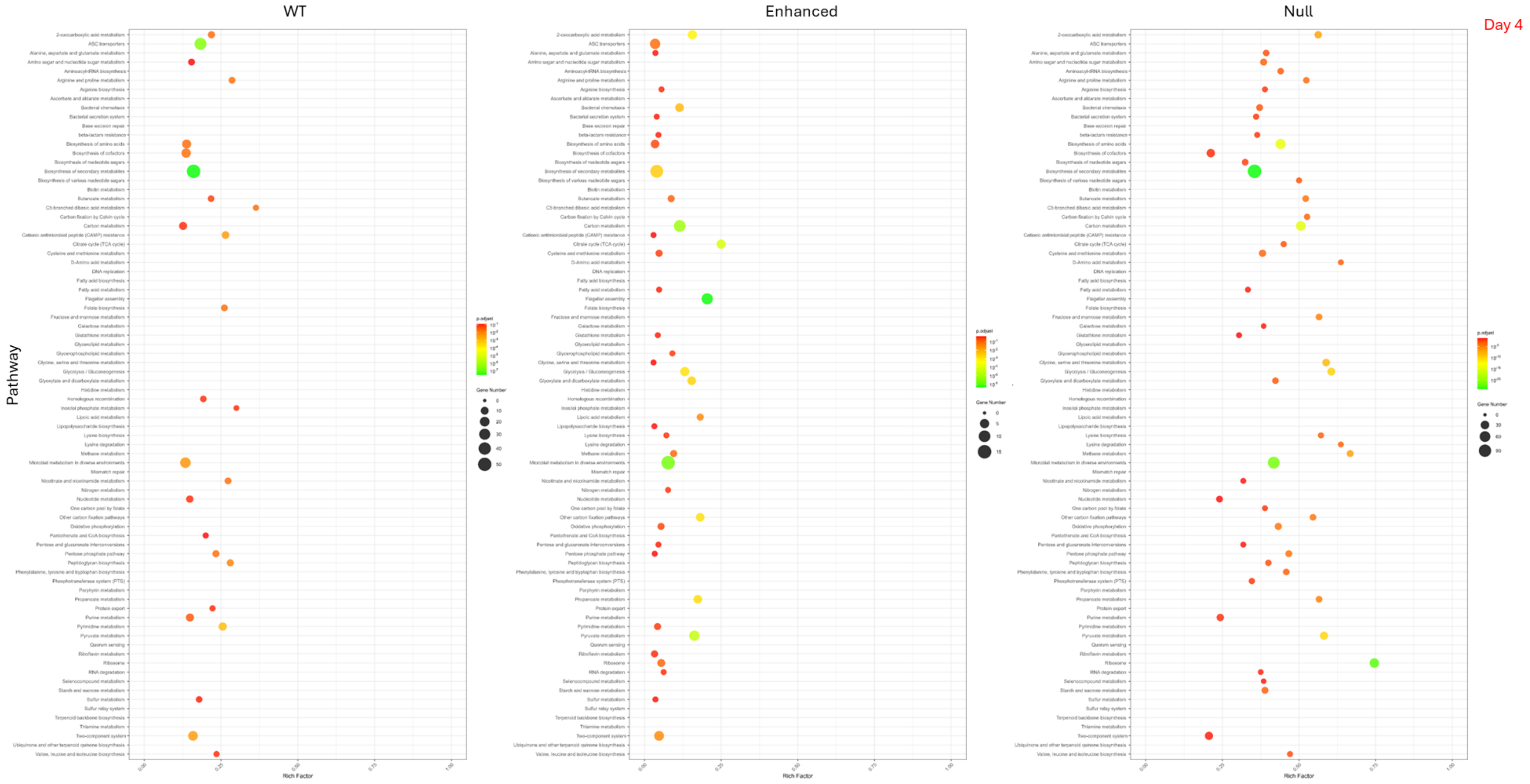

Unofficial

Supplementary 4: KEGG Pathway Enrichment in *Pantoea rara* wildtype and mutant strains on Day 6. The bubble plot shows the results of KEGG pathway enrichment analysis for differentially expressed genes (DEGs) the three strains when PVK and R2A are compared. Pathways are plotted on the y-axis and the x-axis indicates the RichFactor. Bubble size reflects the number of DEGs mapped to the pathway and the bubble colour indicates the statistical significance of enrichment (p-adjust <0.05).

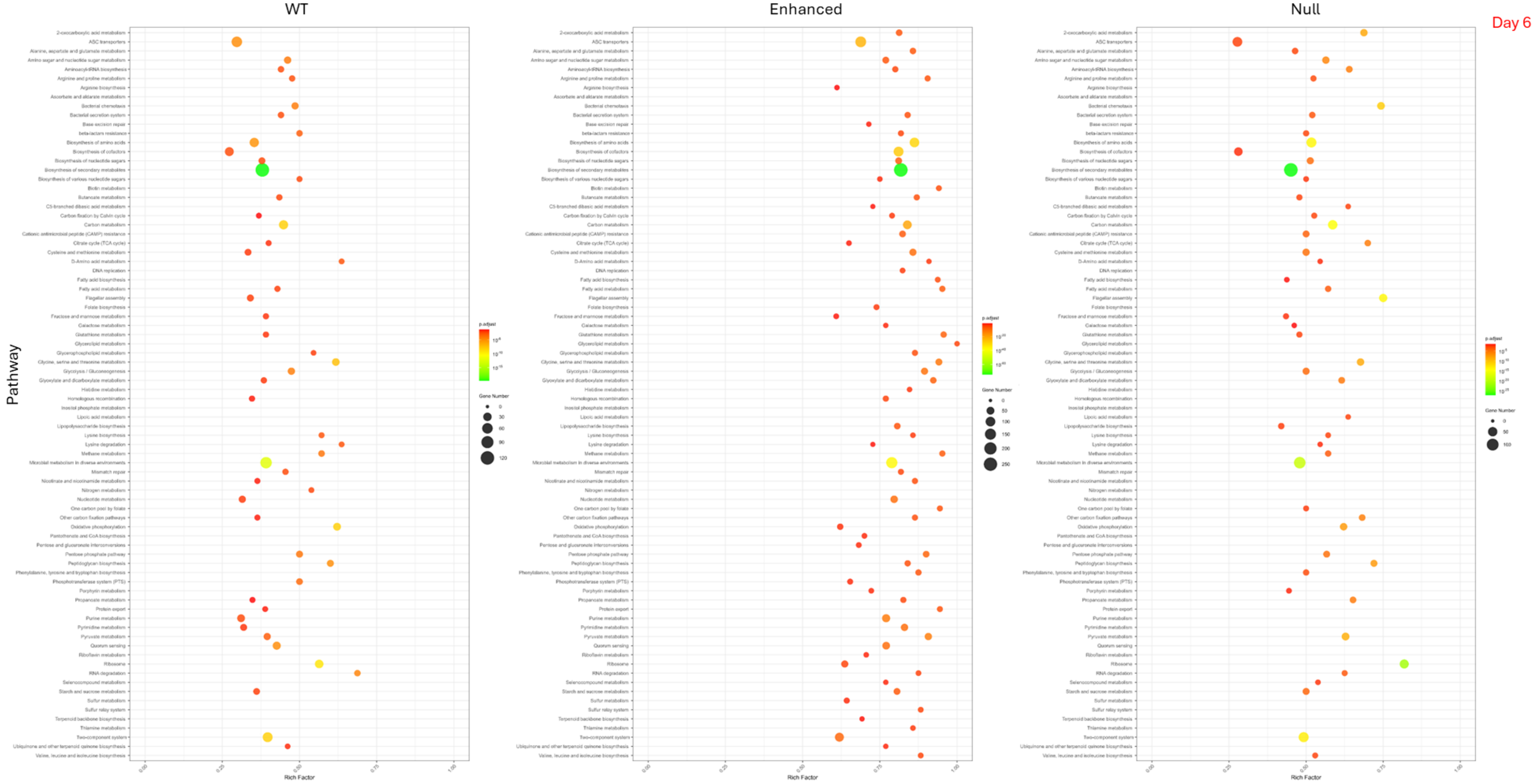

Unofficial

Supplementary 5: KEGG Pathway Enrichment in *Pantoea rara* wildtype and mutant strains on Day 8. The bubble plot shows the results of KEGG pathway enrichment analysis for differentially expressed genes (DEGs) the three strains when PVK and R2A are compared. Pathways are plotted on the y-axis and the x-axis indicates the RichFactor. Bubble size reflects the number of DEGs mapped to the pathway and the bubble colour indicates the statistical significance of enrichment (p-adjust <0.05).

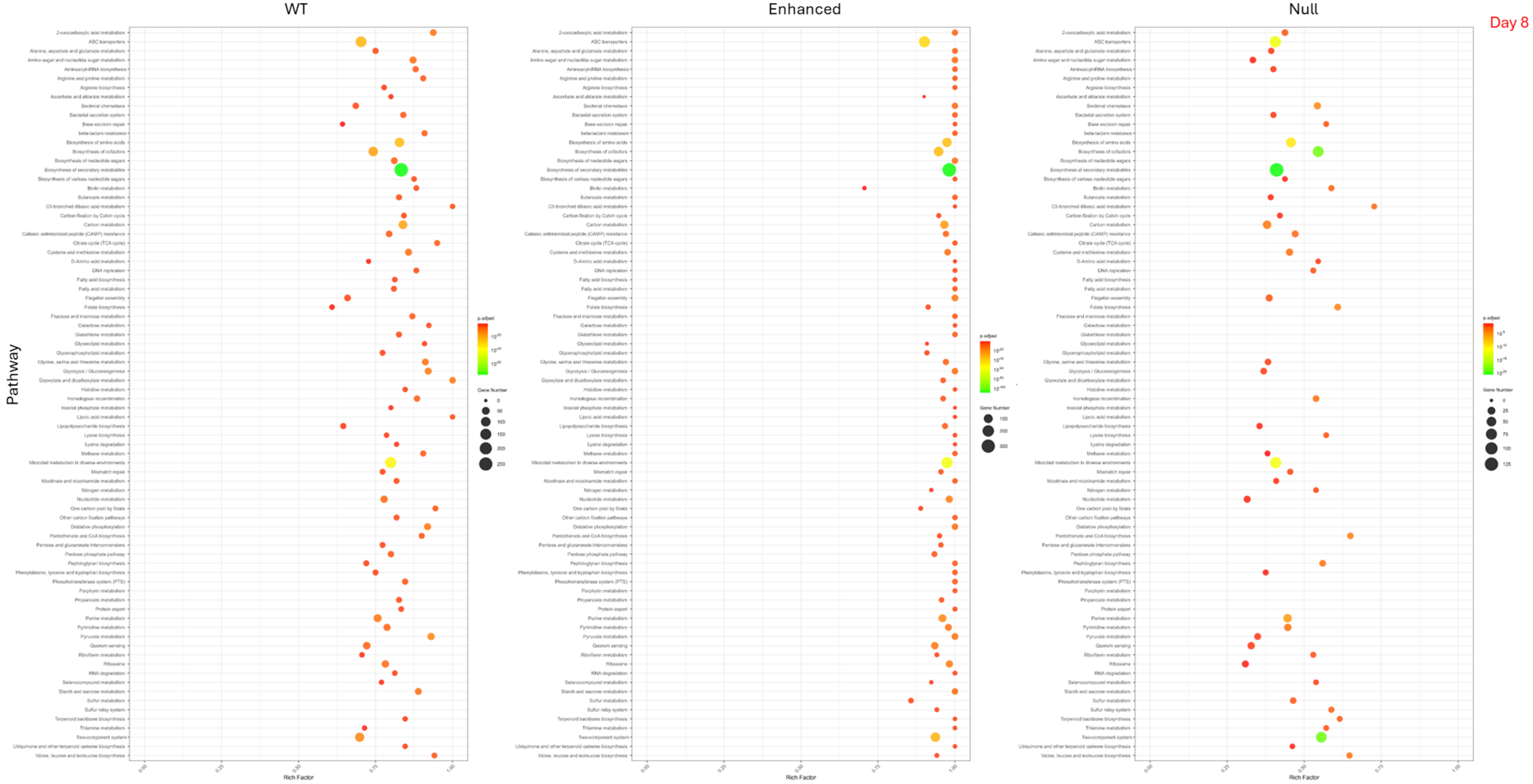

Unofficial

Supplementary 6: KEGG Pathway Enrichment in *Pantoea rara* wildtype and mutant strains on Day 10. The bubble plot shows the results of KEGG pathway enrichment analysis for differentially expressed genes (DEGs) the three strains when PVK and R2A are compared. Pathways are plotted on the y-axis and the x-axis indicates the RichFactor. Bubble size reflects the number of DEGs mapped to the pathway and the bubble colour indicates the statistical significance of enrichment (p-adjust <0.05).

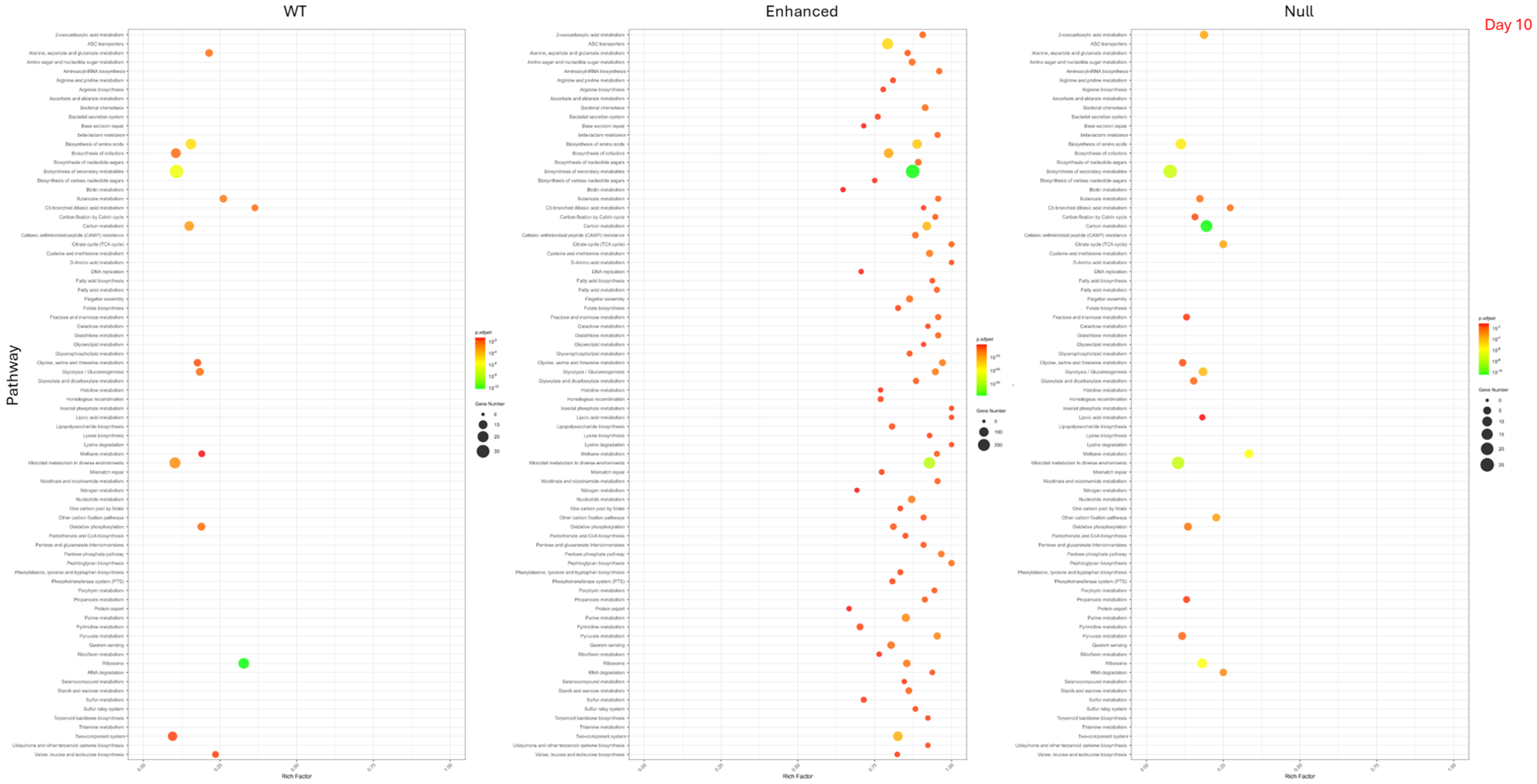

Unofficial

Supplementary 7: Results of PERMANOVA (Adonis) testing based on Bray-Curtis dissimilarities of normalized transcriptomic profiles. The table reports R^2 values (variance explained) and associated p-values for each pairwise comparison of experimental conditions used in the principal coordinates analysis (PCoA).

R2	2day	4day	6day	8day
4day	0.027259	NA	NA	NA
6day	0.078822	0.051421	NA	NA
8day	0.082591	0.062222	0.015875	NA
10day	0.045801	0.043401	0.046349	0.034797

p value	2day	4day	6day	8day
4day	0.622222	NA	NA	NA
6day	0.015	0.174286	NA	NA
8day	0.01	0.07	0.799	NA
10day	0.174286	0.174286	0.174286	0.3075

R2	WT_R2A	WT_PKA	1_2_R2A	1_2_PKA	6_5_R2A
WT_PKA	0.184823	NA	NA	NA	NA
1_2_R2A	0.128532	0.195999	NA	NA	NA
1_2_PKA	0.254243	0.216174	0.194489	NA	NA
6_5_R2A	0.039656	0.170124	0.130845	0.242687	NA
6_5_PKA	0.282745	0.139362	0.225572	0.123235	0.254306

p value	WT_R2A	WT_PKA	1_2_R2A	1_2_PKA	6_5_R2A
WT_PKA	0.001154	NA	NA	NA	NA
1_2_R2A	0.001154	0.001154	NA	NA	NA
1_2_PKA	0.001154	0.001154	0.001154	NA	NA
6_5_R2A	0.368	0.001154	0.001154	0.001154	NA
6_5_PKA	0.001154	0.001154	0.001154	0.005357	0.001154

Unofficial

Supplementary 8: KEGG ascorbate and aldarate metabolism pathway overlaid with differential gene expression profiles of P. rara wildtype and mutant strains. The KEGG ascorbate and aldarate metabolism pathway (map00053) is displayed with gene expression fold change mapped onto the pathway nodes, highlighting how genes are transcriptionally modulated in response to phosphate conditions and UV-induced mutations. Each gene node in the KEGG pathway is coloured according to DeSeq2's Wald test-derived LFC (WT in the first third, Lu_Sq_004_1_2 in the middle third, Lu_Sq_004_6_5 in the last third) in expression of that gene between limited soluble phosphate PVK and replete soluble phosphate R2A at Day 8.

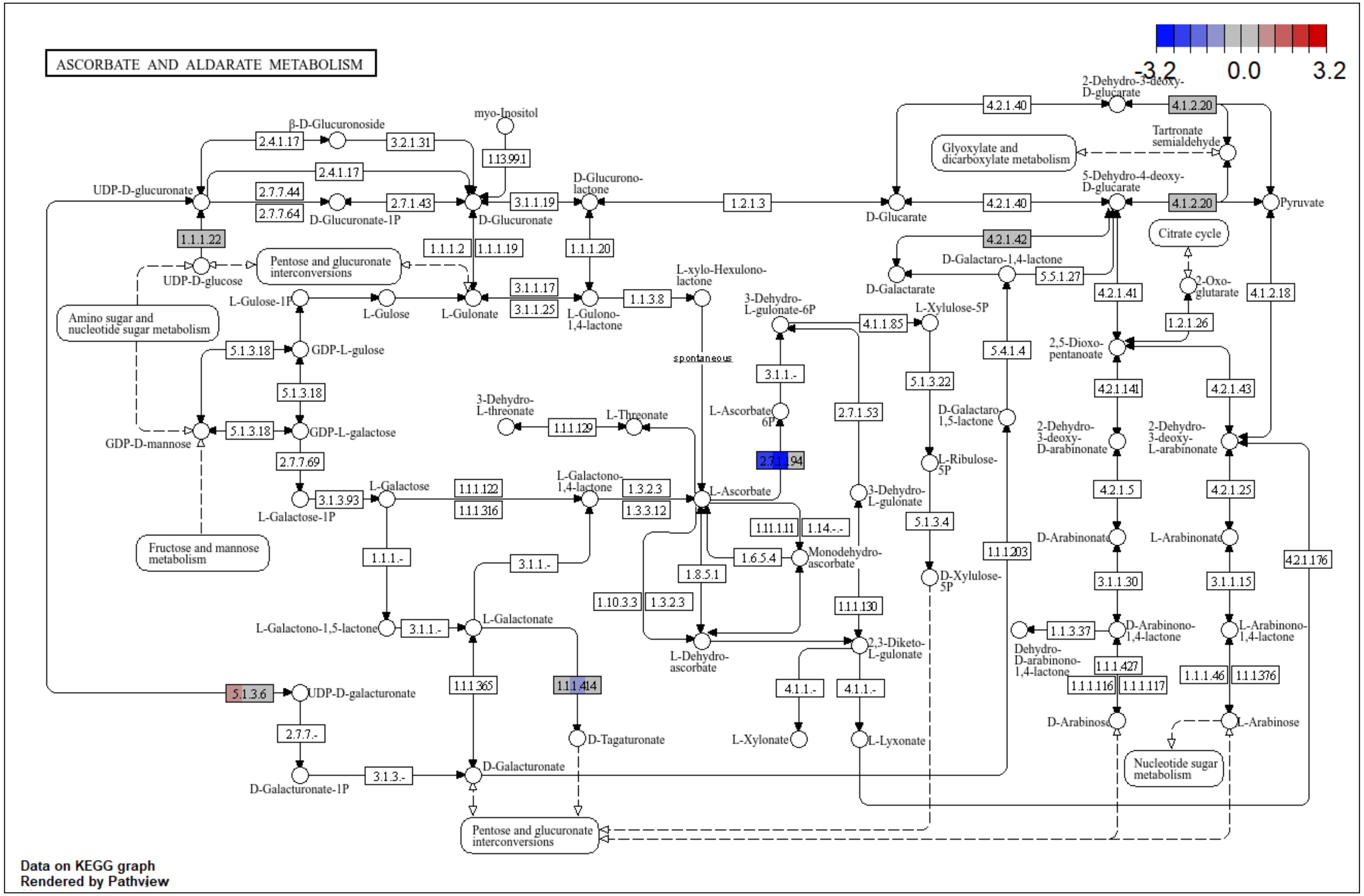

Unofficial

Supplementary 9: KEGG glycerolipid metabolism pathway overlaid with differential gene expression profiles of P. rara wildtype and mutant strains. The KEGG glycerolipid metabolism pathway (map00561) is displayed with gene expression fold change mapped onto the pathway nodes, highlighting how genes are transcriptionally modulated in response to phosphate conditions and UV-induced mutations. Each gene node in the KEGG pathway is coloured according to DeSeq2's Wald test-derived LFC (WT in the first third, Lu_Sq_004_1_2 in the middle third, Lu_Sq_004_6_5 in the last third) in expression of that gene between limited soluble phosphate PVK and replete soluble phosphate R2A at Day 8.

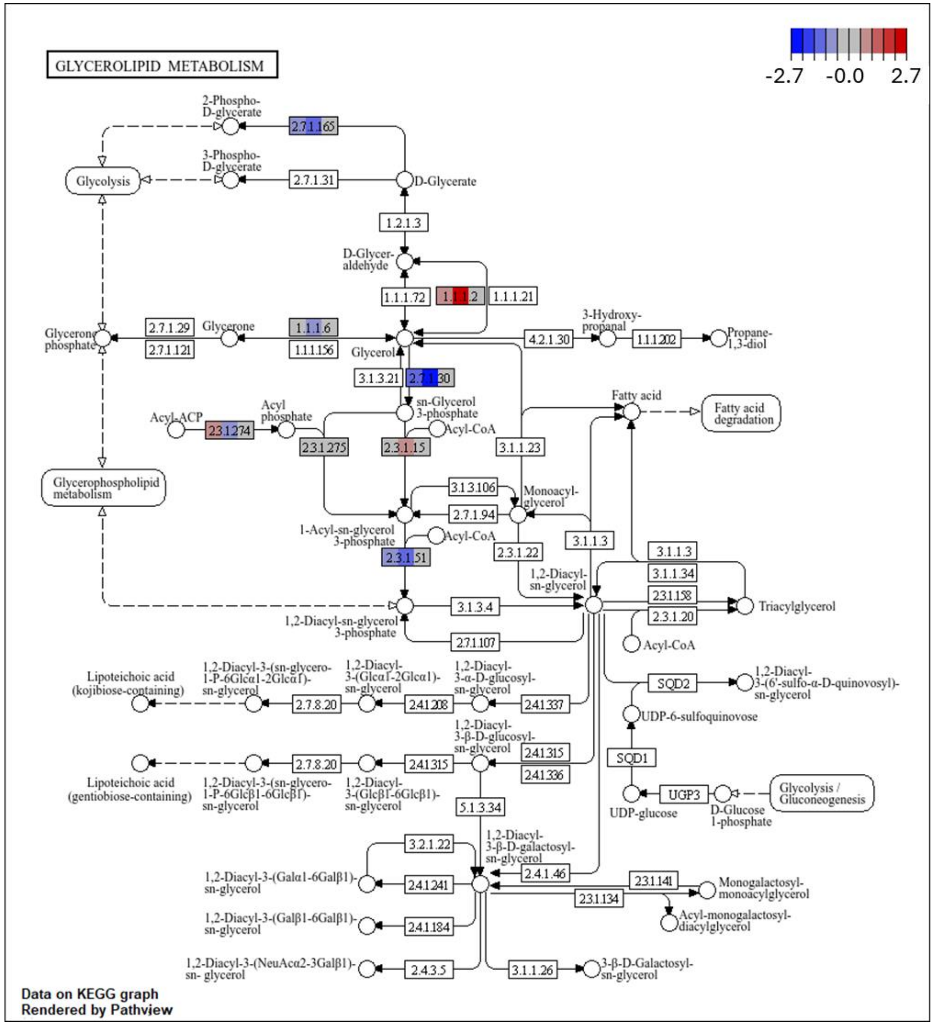

Unofficial

Supplementary 10: KEGG inositol phosphate metabolism pathway overlaid with differential gene expression profiles of P. rara wildtype and mutant strains. The KEGG inositol phosphate metabolism pathway (map00562) is displayed with gene expression fold change mapped onto the pathway nodes, highlighting how genes are transcriptionally modulated in response to phosphate conditions and UV-induced mutations. Each gene node in the KEGG pathway is coloured according to DeSeq2's Wald test-derived LFC (WT in the first third, Lu_Sq_004_1_2 in the middle third, Lu_Sq_004_6_5 in the last third) in expression of that gene between limited soluble phosphate PVK and replete soluble phosphate R2A at Day 8.

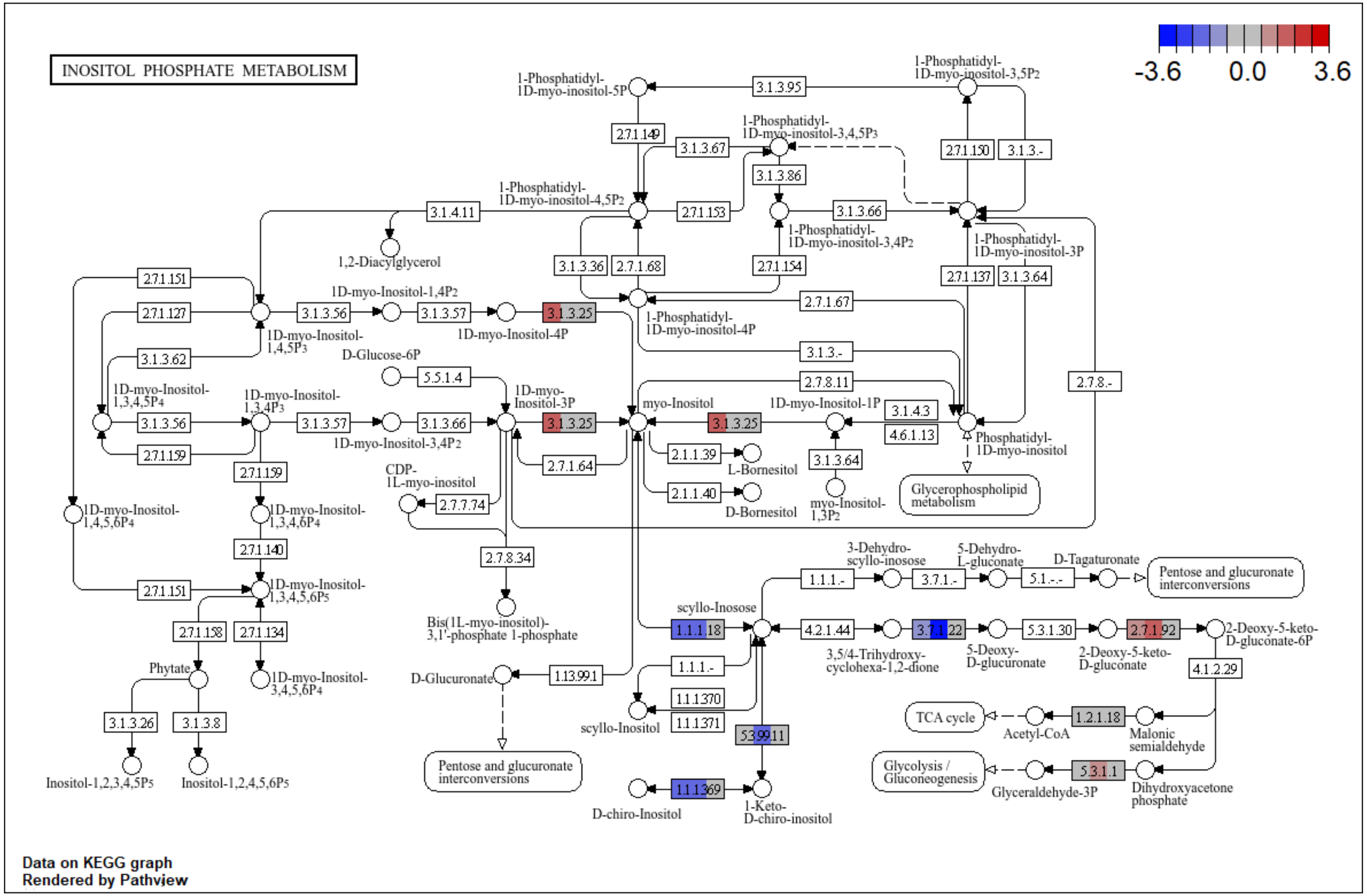

Unofficial

Supplementary 11: KEGG glycerophospholipid metabolism pathway overlaid with differential gene expression profiles of P. rara wildtype and mutant strains. The KEGG glycerophospholipid metabolism pathway (map00564) is displayed with gene expression fold change mapped onto the pathway nodes, highlighting how genes are transcriptionally modulated in response to phosphate conditions and UV-induced mutations. Each gene node in the KEGG pathway is coloured according to DeSeq2's Wald test-derived LFC (WT in the first third, Lu_Sq_004_1_2 in the middle third, Lu_Sq_004_6_5 in the last third) in expression of that gene between limited soluble phosphate PVK and replete soluble phosphate R2A at Day 8.

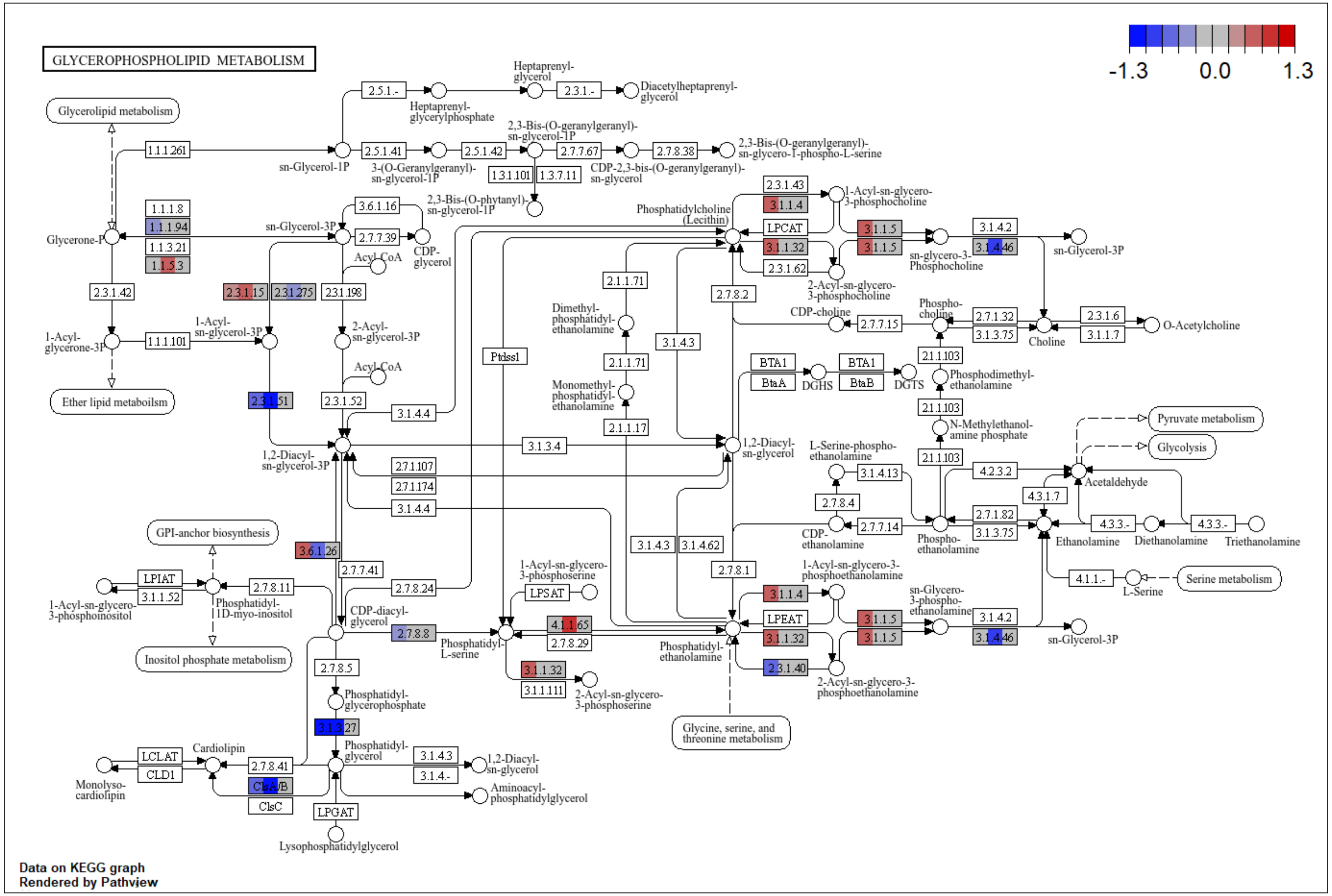

Supplement: Tables S1 to S11 — Tables on strains, bubble plots, and extra KEGG pathways. [file spectrum.03363-25-s0001.pdf]
